# Self-Assembling Hydrogel Structures for Neural Tissue Repair

**DOI:** 10.1021/acsbiomaterials.1c00030

**Published:** 2021-03-29

**Authors:** Sofia Peressotti, Gillian E. Koehl, Josef A. Goding, Rylie A. Green

**Affiliations:** ‡ Department of Bioengineering and ^§^Centre for Neurotechnology, Imperial College London, London SW72AS, United Kingdom

**Keywords:** self-assembling peptides, tissue engineering, neuroengineering, neuroregeneration, peptide synthesis, review, conductive biomaterials, scaffold, bioactive

## Abstract

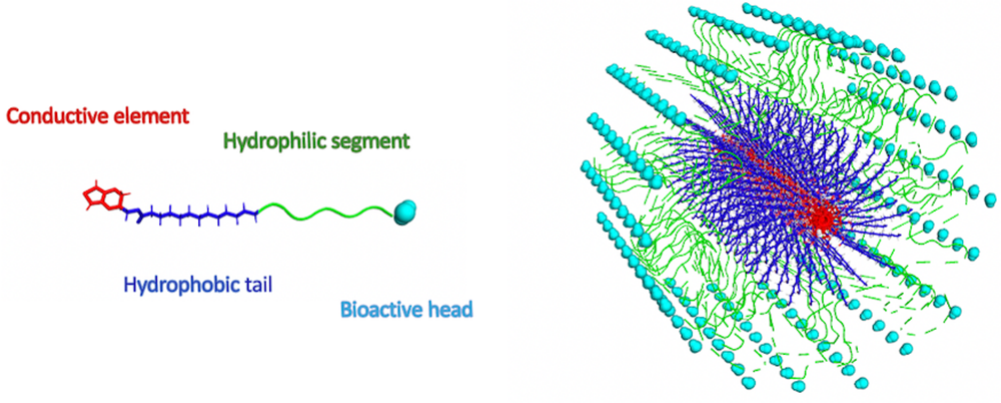

Hydrogel materials have been employed as biological scaffolds for tissue regeneration across a wide range of applications. Their versatility and biomimetic properties make them an optimal choice for treating the complex and delicate milieu of neural tissue damage. Aside from finely tailored hydrogel properties, which aim to mimic healthy physiological tissue, a minimally invasive delivery method is essential to prevent off-target and surgery-related complications. The specific class of injectable hydrogels termed self-assembling peptides (SAPs), provide an ideal combination of in situ polymerization combined with versatility for biofunctionlization, tunable physicochemical properties, and high cytocompatibility. This review identifies design criteria for neural scaffolds based upon key cellular interactions with the neural extracellular matrix (ECM), with emphasis on aspects that are reproducible in a biomaterial environment. Examples of the most recent SAPs and modification methods are presented, with a focus on biological, mechanical, and topographical cues. Furthermore, SAP electrical properties and methods to provide appropriate electrical and electrochemical cues are widely discussed, in light of the endogenous electrical activity of neural tissue as well as the clinical effectiveness of stimulation treatments. Recent applications of SAP materials in neural repair and electrical stimulation therapies are highlighted, identifying research gaps in the field of hydrogels for neural regeneration.

## 1 Introduction

Neural tissue loss represents a complex clinical challenge, which translates to a heavy burden for society. As an indicator of impact, the economic loss has been estimated at $800 billion in the United States alone.^1^ Given the ever growing number of patients suffering from irreversible neural damage due to neurodegenerative diseases, traumatic brain injury, and spinal cord and peripheral nerve injury, a reliable strategy for neural repair and regeneration is a pressing healthcare necessity.^2−5^ The primary challenge in addressing neural tissue loss is its low regenerative capacity, which limits functional recovery after neural injury.^2,6^ Particularly, the injured central nervous system (CNS), which triggers an inhibitory response toward physiological regeneration, hinders functional recovery and promotes the formation of scar tissue.^7,8^ Although the peripheral nervous system (PNS) has more capacity for neuroregeneration, with recovery possible if the damage is relatively minor, larger injuries where nerve bundles must bridge lengths greater than 1 cm have limited solutions for functional recovery.^9−12^ In this context, neuroregeneration refers to a total or partial recovery of tissue functionality by neuronal regrowth or repair, including neurogenesis of the endogenous tissue, physiological repair mechanisms, and exogenous cell transplants.^2^ Considerable efforts have been made toward understanding the underlying mechanisms of neural repair, as well as the development of clinically relevant approaches to encourage neurogenesis, spanning drug development and delivery, tissue engineering, and electrical stimulation strategies.^6,13−17^ Critical to most tissue engineering approaches are biomaterials that act primarily as scaffolds for supporting cell delivery and growth but can also be used for drug delivery and provision of electrical stimuli.

The overarching aim of tissue engineering scaffolds is to use a material system to mimic the physicochemical properties of the natural tissue milieu.^18,19^ Biomimetic scaffolds, made from biologically inspired materials, provide environmental cues that target desired biological mechanisms.^20,21,254^ Such biomimetic cues can be used to control cell and tissue behavior, promoting neural tissue regeneration and repair. These elements can take the form of bioactive molecules and pharmaceuticals, as well as mechanical and topographical cues for physical support.^6^ These tissue scaffold materials need to be carefully designed and tailored to elicit the desired cellular responses and thus provide a therapeutic effect.

Hydrogel systems are the most commonly applied biomaterial for soft tissue engineering. Hydrogels are ideal for these applications because of their structural and mechanical similarity to the extracellular matrix components, their general cytocompatibility, and their capacity to provide biological cues.^22,18,21,23−26^ A variety of hydrogel materials have been investigated for neural applications, spanning from natural tissue components to entirely synthetic materials.^26,27^ Biologically sourced materials including acellularized tissue and extracellular matrix-derived macromolecules such as collagen, chitosan, and hyaluronic acid have been used extensively. They are advantageous because they are nontoxic, cytocompatible, simple to obtain, and have inherent bioactive cues, however biologically sourced materials carry a risk of immunogenicity and may be prone to batch-to-batch variability.^28^ Synthetic polymers present an alternative with significant benefits, including reproducibility and versatile tailoring through simple modifications of pendant groups. Common examples used in tissue engineering constructs include poly(ethylene glycol) (PEG), poly(vinyl alcohol) (PVA) and poly(ethylene oxide) (PEO).^29−31^ However, these purely synthetic hydrogels lack critical biological cues, limiting their biomimetic properties.^28,32^ As such, tuning of the physicochemical properties and biofunctionalization of these polymeric materials toward a more biomimetic material is often necessary. Biologically inspired proteins or polymers are a third class of material that provides a higher degree of control in contrast to biological polymers, but being based on natural amino acids (AAs) can be assembled to incorporate critical biological cues, such as adhesion sequences.^33,34^ These synthetic peptides can be cross-linked into tunable, nontoxic, and biofunctionalized hydrogels, making them a promising material choice for neuroregeneration applications.^26,35−37^

Cell scaffolds are intended to physically support the surrounding tissue during regeneration. Historically, the scaffold shape and size were defined preimplantation, leading to surgical invasiveness and long recovery periods.^23,38,39^ This was due to the need for material polymerization and implant definition prior to the surgery as a means of controlling the polymer structure and structural features.^40,41^ The more recent development of minimally invasive and in situ surgical approaches has fostered the development of injectable systems.^42^ These systems have found utility in neural repair, as they support localized treatment and minimize postsurgical complications, demonstrating versatility for translation to the clinic.^6,20^ Injectable materials permit the formation of a hydrogel *in situ* via the minimally invasive delivery of a hydrogel precursor to the desired location. Once injected, the hydrogel can be formed using a variety of physical or covalent cross-linking methods, including environmental stimuli such as temperature, pH and salt concentration.^20,42−45^ Both natural and synthetic polymers can be designed to be injectable, such as chitosan-based thermoresponsive hydrogels or injectable PEG polymers.^46,47^ The combination of hydrogel precursor and method of polymerization will determine the final molecular arrangement, allowing for finely controlled macromolecular conformations.^48,49^

The class of injectable materials termed self-assembling, offer a thermodynamic advantage by exploiting spontaneous physical interactions of the molecules in the environment, forming stable network microstructures.^50^ The design of self-assembling polymers requires a precise understanding of chemical structures and molecular interactions that impact on the assembly mechanisms from monomer or macromonomer into a hydrogel network.^6,32,51^ The addition of biofunctional groups must not chemically or structurally interfere with the self-assembling cross-linking mechanism of the polymer, and simultaneously the mechanical and structural properties need to be maintained within the physiological range.^20^ This complex design challenge requires versatile control over the polymer chemical and structural composition. Among all material types, peptide-based polymers offer the possibility to easily implement self-assembling mechanisms by mimicking natural aggregation processes, while maintaining the required physicochemical properties.^52−54^ The synthetic peptides that spontaneously assemble into ordered nanostructures under physiological conditions are named self-assembling peptides (SAPs).^55−57^ One of the major advantages of SAPs among other material types is their simple functionalization with adhesion molecules and their highly biocompatible components. SAP building blocks are effectively single AAs, which are an important component of the physiological environment.^19,55^ Besides the simple synthesis, functionalization and property modification, these materials allow for minimally invasive treatments, which are critical in neural injury or disease.^55−58^

This review examines the recent developments in SAP systems designed for neural applications, including methods to tailor SAP properties to optimize their performance as neural scaffolds which can guide neural repair. Key design criteria are identified from an overview of the physiological tissue properties, with the aim of replicating the main features of the neural environment within the biomaterial. Ways to control and tailor properties of SAP constructs, such as self-assembling mechanisms, mechanical properties, topography, and bioactivity are considered as biomimetic cues through the lens of cell–material interactions. Furthermore, the incorporation of conductive scaffolds and electrical stimulation within SAP constructs to promote neural regeneration is assessed. Finally, the latest SAP-based applications for neural regeneration are presented, to identify their advantages and limitations, highlighting the latest technological advances and unmet clinical needs.

## 2 Biomimetic Cues for Neural Repair

Cells need to sense specific biomimetic cues expected from the native ECM and healthy neural tissue to accomplish neural regeneration and repair. It is essential to consider these requirements for neural repair in the design of materials systems intended to address neural injury. Materials used for neural repair should therefore aim to mimic the neural environment with finely tuned physicochemical properties engineered to interact with the target cell types and tissue features.^21,59^ Understanding the specific injury environment that a biomaterial is intended to address is critical to the successful development of an injectable neural scaffold. The functionality and structure of physiological neural tissue relies on the synergy between a multitude of specialized cell types and a complex microbiological milieu. For instance, the CNS and PNS have different responses to injury and vary in their potential for regeneration.^60−63^

After a peripheral nerve injury, the distal segment of the axon undergoes an initial degeneration that inhibits growth in the initial stage, followed by the secretion of neurogenic signaling pathways by Schwann cells and the formation of growth cones for functional nerve regeneration.^64^ Conversely, the injury setting in the CNS triggers the reaction of microglia, astrocytes and oligodendrocytes, which inhibit regeneration and promote the creation of a glial scar.^7^ The two conditions present a different biochemical environment, characterized by specific ECM composition, signaling cues and cell types. Design of a biomaterial implant should consider all the relevant components and create a favorable environment for the proliferation, development and neurogenic behavior of target cell types. Drugs and bioactive molecules can also be incorporated within a material system for a multifunctional therapeutic approach.^6,65,66^ Biomaterials and in particular hydrogels may also be used as cell carriers in stem cell transplants to control cell fate and promote neuroregenerative processes.^20,39,67^ The material cues for this application should replicate the neural stem cell (NSC) niche, a biophysical microenvironment that regulates differentiation cues and cell fate.^68^ Cues toward neuronal lineage, as opposed to glial and epithelial, are preferred for an optimal integration with the endogenous nervous system.^69,70^

The design of biomaterials targeting neuroregeneration should account for the complex host–material interactions for specific injury environments, tailoring the cell interaction to the targeted tissue type, diseases environment and cell type.^71,72^ Specific design criteria for material parameters and composition should be defined by considering the key components of the native neural milieu and their effect on cell behavior. Among all material types, injectable materials require extremely precise tuning and characterization of the biomimetic features postassembly, because the in vivo polymerization does not allow for a preimplantation control of the material properties and self-assembling bioproducts. Fundamental material features such as mechanical properties, degradation mechanisms, biochemical composition, structural features, and conductivity should be investigated in light of both the physiological environment and the cell–material interactions to define effective injectable material properties and modifications.

### 2.1 Biological Cues

The primary requirement for neural repair is the presence of a biochemical environment that supports neural cell populations.^18,27^ Cell behavior can be directed toward neuroregeneration through the incorporation of bioactive cues within biomaterials.^21,24−26,73,74^ This material modification is exceptionally important in neural applications, given the low inherent low regenerative potential of this tissue type.^75^ The native ECM offers essential biochemical and structural cues to neural cells, which sense the environment through adhesion molecules, termed integrins.^76−80^ Integrins are specialized adhesion receptors that interact with peptide sequences present in the ECM and regulate cell–cell interactions.^81^ They interact with the cell cytoskeleton and influence gene expression, proliferation, and survival through bidirectional signaling with the biochemical environment.^81−84^ It follows that the presence of integrin-binding factors is a paramount design requirement in biomaterials. Specifically, this includes ensuring cell adhesion through the presence of naturally derived materials or the presence of biomimetic adhesion molecules.^79−81,84^

To inform the design of bioactive cues within hydrogels for neural repair, it is key to examine the native ECM components, which provide the necessary factors for healthy cell growth and differentiation. The brain ECM is a complex meshwork of multiple compounds. Aside from typical ECM components such as collagen, laminin, hyaluronic acid, and fibronectin, the brain ECM is extremely rich in glycosaminoglycans (GAGs), including chondroitin sulfate and hyaluronan.^85,75,86,87^ Chondroitin sulfate influences neural plasticity and cell behavior through sequences of sulfate groups on the GAG molecule backbone, conveying functional information through sulfation codes.^75,88,89^ In the case of the PNS, laminin, and collagen are fundamental ECM components for their role as Schwann cell regulators.^90^ It follows that ECM adhesion molecules are considered a powerful tool to direct cell behavior.^79−81,84^ In neural scaffolds laminin, collagen and hyaluronic acid are often selected as adhesion substrates in their natural or synthetic form.^84,87^ In particular, laminin-derived peptides in neural cultures are able to increase neural cell migration, proliferation and differentiation toward neuronal fate.^84,91,92^ Short bioactive sequences of AAs involved in the adhesion signaling, termed bioactive epitopes, are often exploited as adhesion cues in tissue engineering. The bioactive epitopes contained in laminin, collagen and fibronectin molecules, including RGD, IKVAV, and YIGSR, are the most widely used examples^93^ (the reader is referred to Koss and Unsworth;^58^ see Table 2 for a comprehensive review of adhesion molecules for neural regeneration). Moreover, GAGs such as chondroitin sulfate represent an effective element to introduce into a bioactive scaffold for the central role in the neural ECM.^94−96^ Adhesion molecules and their effect on neural cells should be carefully selected from the neural ECM components, tailoring the material composition toward the targeted regeneration application. Given the complexity of the natural biochemical milieu, replicating the biological cues in a material system is a design challenge. Often, hydrogel materials can be functionalized with a relatively small number bioactive molecules because of the low availability of chemical bonds that can be formed without affecting the self-assembling mechanism and molecular interactions.^97,98^ A trade-off between bioactivity and hydrogel stability and structure must be achieved.^23,40^

Aside from adhesion peptides, other bioactive molecules such as growth factors (GFs), cytokines, and signaling molecules are considered effective cues acting through regeneration-related molecular pathways in the CNS and PNS.^75,89,99^ GFs are a widespread class of proteins that can stimulate cell growth, differentiation, and wound healing.^2,23,100,101^ Cell-binding of GFs activate intracellular second messenger systems through cell surface membrane receptors that affect neural cell growth and differentiation.^58,100,102,103^ GFs are produced by healthy cell populations and can direct NSC differentiation toward specific cell types.^74^ Nerve growth factor (NGF), brain-derived neurotrophic factor (BDNF), and tyrosine kinase (Trk) are important examples of a neurotrophic factors involved in neural development which enhance neuronal differentiation.^100,102,103^ Other methods of biochemical guidance include signaling molecules that drive gene cascades toward neural repair or differentiation.^104,105^ For instance, the delivery of a molecule dubbed TTK21 was recently proven to promote spinal cord regeneration and sprouting of sensory and motor axons through epigenetic reprogramming.^104,105^ In addition, the neural chemical signaling molecules neurotransmitters are known to influence neural plasticity and are involved in strengthening neural connections and glial cell stimulation.^28,106,107^ GFs and bioactive molecules can be incorporated in the material system to enhance neural regeneration or direct cell fate, and their effect can be tailored for regenerative or drug delivery applications.

### 2.2 Mechanical Properties

**Figure 1:**
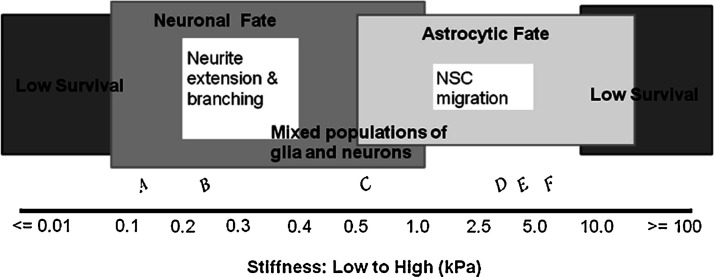
Effect of material stiffness on neural stem cell fate in vitro. A stiffness of around 1 kPa allows the presence of a mixed neural population, whereas excessively high or low values decrease cell survival. Reproduced with permission from ref (18). Copyright 2012 Elsevier.

The mechanical properties of neural tissue vary depending on tissue type and location. In general, the brain has a low stiffness and presents viscoelastic properties, whereas nerves and the spinal cord show higher tensile strengths due to the alignment of the nerve fibers.^108−110^ More comprehensive properties of the brain, spinal cord, and PNS are presented in Table 1. The importance of biomimetic mechanical properties for neural cells is largely related to the mechanotransduction of key biological signals.^6,21,24,111^ Transmembrane proteins, primarily integrins, are intrinsically mechanosensitive and affect cell behavior and growth depending on substrate stiffness^18,83^ (Figure 1). Binding of these transmembrane proteins allows the mechanical signal to be converted into downstream chemical pathways, which are known to affect cell adhesion, morphology, and differentiation.^93,112,113^ For example, at stiffnesses comparable to physiological neural tissue (100–500 Pa), NSC differentiation can be directed toward a substantial neuronal subpopulation as opposed to astrocyte and oligodendrocyte populations.^114−116^ Koser et al.^114^ showed that axon length and degree of spreading varies with substrate stiffness. Softer substrates were shown to encourage more exploratory growth, better suited for synaptic formation, whereas stiffer substrates promoted faster, straighter, and more parallel growth of axons.^117^ A stiffness range above 200 kPa can lead to apoptotic activity and reduced viability of in vitro neural cultures.^93^ This phenomenon has also been observed in the clinical setting, where damage to the CNS causes the formation of scar tissue, or glial scar, which dramatically increases the stiffness of the tissue, leading to neural loss and cell death.^7,8^ Zhong et al.^118^ have performed a comprehensive review of mechano-sensing under 2D and 3D environments.

**Table 1:** Mechanical Properties of Brain, Spinal Cord, and PNS Tissue

	Young’s modulus	compressive modulus	properties
brain	40 to 20 000 Pa, human^108^	3–6 kPa, rat^116^	nonlinear viscoelastic behavior^108^
	23.8 ± 10.5 kPa (50/s strain rate)	3.4 kPa, embryonic rat forebrain^122^	
	38.5 ± 2.0 kPa (60/s strain rate), porcine^121^		
	3–10 kPa Young’s (elastic) modulus, human^123^		
spinal cord	0.3–1.4 MPa, human^109^	8.1 ± 1.1 kPa, adult rat^116^	
PNS	1.2 MPa, mice lumbar nerve roots		
	7 MPa, mice sciatic nerve^110^		

When designing a biomaterial scaffold for neural repair, the mechanical properties should be based on physiological ranges, and design criteria should specifically target substrate stiffness to support neuron survival and direct cell behavior toward regenerative processes. Modifications of the elastic or compressive modulus can be implemented in material systems to match the target tissue features with relatively simple approaches that have been detailed in the literature.^30,119,120^ However, an engineering challenge can be identified in the design of injectable materials. Self-assembly mechanisms can be affected by variable physiological conditions and delivery methods, such as temperature, chemical composition of the target site, or injection speed.^43^ These features can cause difficulties in achieving precise mechanical properties to ensure physical support to the cells.^42,47^

Importantly, mechanical support provided to encapsulated cells changes dynamically with material degradation, which can be tuned to match natural tissue growth.^39,124^ Neural cells interact with their environment by degrading as well as producing ECM.^18,30^ Neural tissue physiological remodelling is a fundamental process in healthy tissue environments, involved in tissue turnover, synaptic plasticity and neural repair. Enzymes known as matrix metalloproteinases (MMPs) are responsible for ECM degradation and remodelling and promote tissue growth and differentiation.^125^ Neurons and glia secrete degradation MMPs and contribute to ECM remodelling in physiological conditions, brain injury, and other brain disorders such as cancer.^126,85,127−129^ Abnormal ECM dynamics, commonly present in injured or pathological tissue, may also cause imbalances in cell behaviors leading to immune and inflammatory response activation, which encompass the initial stage of spontaneous neural repair. For example, after spinal cord injury (SCI), the molecules released from damaged ECM can trigger and amplify the inflammatory response. The subsequent alterations of the ECM structural and chemical composition affect cell migration, communication, and survival toward a spontaneous regenerative response.^85^ These mechanisms affecting tissue remodelling can be replicated to provide both endogenous and exogenous cells with a substrate to degrade while proliferating and secreting new ECM.^18,130^ A balance between providing mechanical support and allowing space for tissue growth is a central requirement to achieve a physiological cell response to the biomaterial and avoiding adverse responses.^125,131^

The ideal scaffold provides initial mechanical and biochemical support to cells, and its degradation rate should match the ECM formation such that it allows for the regeneration and growth of the new tissue.^18,21^ A trade-off between controlled degradation and biocompatibility should be considered.^18,36^ A high degradation rate can lead to the accumulation of chemical degradation products, which in turn can encourage glial scarring and immune/foreign body response.^18,128,132,133^ Thus, the material composition and degradable chemical bonds should be engineered to match the natural tissue degradation rate of 2–6 weeks.^134^ Degradation is typically due to hydrolytic or enzymatic degradation.^135,136^ Functional groups such as MMP cleavable peptide linkages can be inserted into a biomaterial to match the degradation with local cell proliferation and metabolic activity.^137^ It is important to note that the degradation rate *in vitro* and *in vivo* can vary considerably because of the changes in environmental conditions.^18,138^

### 2.3 Architecture and Topography

The micro- and macroscale structures of neural tissue are linked to their physiological function.^139^ In the PNS, aligned nerve fibers are organized in fascicles depending on function, displaying a hierarchical architecture,^140^.^141^ The nerve sheath, composed of myelin and connective tissue, surrounds and insulates nerve fibers.^141^ The spinal cord has a similar aligned architecture, showing ascending and descending neurons organized in bundles, around 8–60 μm in size^123^.^142^ The brain structure is more homogeneous, with the white matter composed of aligned myelinated nerve fibers and the gray matter consisting of cell bodies and unmyelinated axons, with highly anisotropic structures.^139^ The brain ECM includes perineuronal nets (PNNs), which show lattice-like chondroitin sulfate structures around subpopulations of neurons. They act as growth and migration inhibitors to maintain the tissue structure.^75^ Replicating these physiological structures can be advantageous for a scaffold’s efficacy, given that the tissue architecture can directly affect cell behavior and function.^139^ Indeed, aside from sensing the substrate’s stiffness, surface and adhesion receptors can also respond to the architecture and topography of the environment.^63^

The spatial arrangement of micro- and nanoscale material features can influence cell adhesion, spreading, alignment, and morphology which in turn can alter cell behavior and gene expression.^93,143−148^ It is important to note that historically the majority of *in vitro* cell studies have been performed in 2D cultures.^149,150^ However, the native neural milieu and its physicochemical features are 3D. This implies a significant difference in the way cells are affected by environmental cues. The spatial distribution of the cues is more homogeneous and this affects cell attachment and shape toward a more biomimetic model.^151−153^ As a result, 3D spatial features of a construct can influence the neural cell response, and in vitro 3D cultures created by encapsulating cells within a biomaterial are a preferable method for replicating the neural environment.^149^ 3D architectural cues can be introduced into the material system as topographical cues to neural cells. Topographical cues include every spatial feature and physical modification of the microenvironment, spanning from fibrous structures to roughness of the surface.^80,154,155^ Curtis et al.^156^ have reviewed how cells sense physical features of the environment at the nano- and microscale such as physical patterning, roughness, pits, grooves, and fiber alignment. Surface patterning and roughness affect cell attachment and migration,^157,158^ while chemical patterning modifies cell morphology.^159^ Aligned topography is found to be among the most effective in neural tissue regeneration, due to their polarized morphology, which mimics physiological patterns in neural tissue.^28,32,63,93,143,160−163^ Human NSCs are shown to differentiate toward the neuronal lineage when exposed to aligned microscale patterns, and neurite outgrowth can be enhanced by contact guidance.^93,145,164−166^ For example, dorsal root ganglia cells increase the maximum length of their neurites by 82% when exposed to core–sheath nanofibers.^167^ Baranes et al.^168^ showed that nanotopographies altered gene expression profiles of primary neurons isolated from medicinal leaches, upregulating axon-guidance signaling pathways, synaptogenesis and synaptic regulation, resembling the behavior of interconnected neurons. Human embryonic stem cells (hESCs) can be differentiated into a neuronal lineage by exposing them to an aligned ridge pattern, without the need for other differentiation-inducing agents.^143^ Similarly, human induced pluripotent stem cells (hiPSCs) can be differentiated into neuronal lineages when exposed to aligned microgrooves.^169^ This property can be exploited as a powerful method to control and tune the development of a neural progenitor cell population, and guide its growth at the same time.^170,171^ This cellular response is highly desirable for neural regeneration, and methods to create a material that elicits this cellular response in clinical applications are of utmost interest.^10,172^

Micro- and nanoscale structures can also influence local homeostasis by affecting the accessibility of soluble nutrients, ions and molecules, as well as tissue vascularization.^173^ Specifically, the porosity and pore size of the material should be tuned to allow for molecular diffusion while providing a stable structure for cell growth and proliferation.^173,174^ In neural applications, the pore interconnectivity is essential for neurite growth, with a desirable porosity of 90% and a suitable pore size pore size ranging from 10 to 100 μm.^123,62,173,175−177^

### 2.4 Conductive Properties

Neural cell behavior and growth can be substantially impacted by electrical cues, which are a widespread strategy for neuroregeneration treatments such as nerve repair.^178^ Endogenous electric fields are known to be present in neural development and would healing.^179,180^ Spontaneous activity in the CNS plays a role in the assembly of developing neural circuits, and axon regrowth is promoted by the electrical potential physiologically generated in the wound environment.^179^ Endogenous electrical signals consist of polarized ion transport within the biological tissue, which influences cell membrane potential and electrophysiological state.^180,181^ The conductive properties of different types of neural tissue are presented in Table 2.

**Table 2:** Conductive Properties of the Neural Tissue

Brain (S/cm)	Spinal Cord (S/cm)	PNS (S/cm)^a^
2, whole skull^182^	60, white matter, longitudinal^183,184^	9.1 inside nerve^185^
0.7, inner compact^182^		
0.5, outer compact^182^	8.3, white matter, transverse^183,184^	15.9 epineurium^186^
47, gray matter^182^	23, gray matter^183,184^	57.1 endoneurium longitudinal^186^
		8.3 endoneurium transverse^186^

a Data for the PNS were derived from nerve resistivity values.

Signaling pathways influencing the cell cycle, ion channel expression, and other gene cascades leading to proliferation, migration, and differentiation are activated by electrical activity.^181,187,188^ In the context of neuroregeneration, neuronal guidance through biomimetic electrical signals is a powerful tool to repair nerve and spinal cord injuries.^189−192^ The electrophysiological state of the stem cell niche is known to promote differentiation toward neural lineage and increased neural proliferation.^189,190^ The use of electrical cues in tissue engineering is extensive and spontaneous electrical potentials are a central element for neural development, thus the conductivity and electrochemical properties of scaffold materials used for neural repair are worthy of consideration.^152,191,193−195^ An ideal material will support the endogenous or exogenous electric field propagation to favor neural regeneration.^193,194^ In the context of SAPs, it is essential to ensure the compatibility of the self-assembling physiochemical mechanism with the propagation of electrical signals.^191,196^ Alternatively, electroactive scaffolds can be developed to actively promote electrical stimulation or exposure of cells to electric fields.^189^

**Figure 2:**
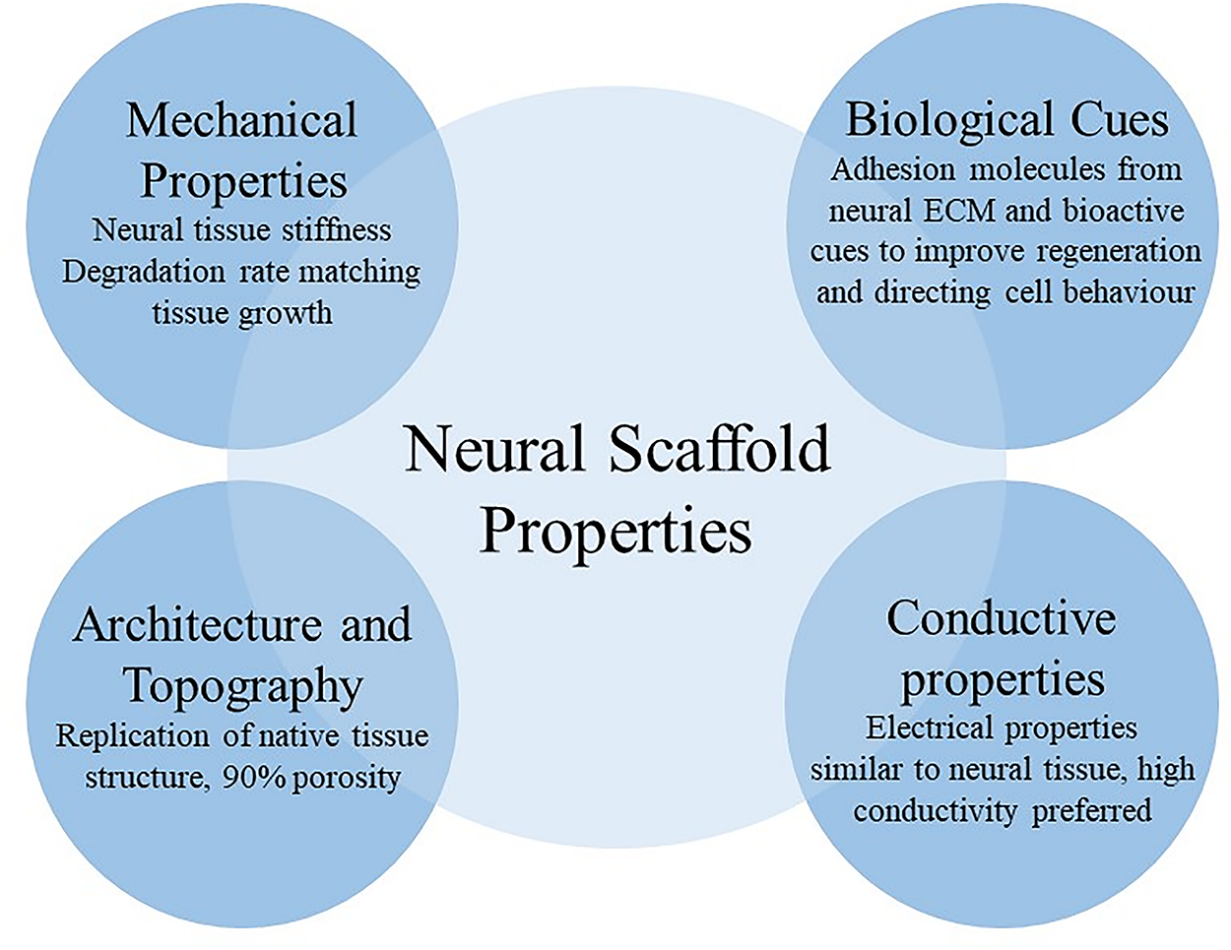
Design criteria for a neural scaffold can be divided into four categories: biological cues, mechanical properties, architecture and topography, and conductive properties.

The reviewed design criteria cover an extensive range of material properties and relative cell–material interactions involved in neuroregeneration mechanisms (Figure 2). Bioactive cues ensure cytocompatibility and direct cell behavior, whereas mechanical properties ensure cell adhesion and proliferation through mechanotransduction. The scaffold topography can guide cell migration and differentiation. Lastly, conductive properties of the scaffolds allow the compatibility of hydrogels with stimulation treatments as well as supporting spontaneous electrical activity. A close investigation of the native neural environment is crucial and largely encouraged for defining material design criteria as well as fostering novel bioinspired hydrogel systems, toward a multifunctionalized highly effective self-assembling material.

## 3 Self-Assembly Biomaterials for Neural Repair

Scaffolds for tissue engineering neural repair should minimize invasiveness and provide topographical, structural, biomechanical and biochemical support for neural regeneration. Various attempts have been made using synthetic or biological materials; however, all these material modifications need to be considered with regards to their potential impact on the physicochemical properties of the biomaterial construct.^60^ Facilitating topographical cues via injectable materials is challenging as it typically requires *in situ* formation of structural elements. Self-assembling materials enable the formation of various topographies upon injection in vivo due to their responsiveness to local environments. Therefore, careful design of the material can lead to control over physiochemical properties in order to achieve a scaffold that meets the criteria for neural regeneration.^197^ Self-assembly is governed by supramolecular chemistry as it relies on non-covalent forces between molecules. It is therefore important to understand the forces that govern the self-assembly process in order to tune the assembled structures and their properties for a specific application. Methods have been developed to tune the topographical, mechanical, bioactive and conductive properties of self-assembling materials. The application of these methods to SAPs can be tailored to create a biomimetic and effective material support.

Noncovalent interactions between molecules are the driving force for the spontaneous formation of organized structures, a process called self-assembly that occurs readily in nature at various length scales. A variety of molecular driving forces can be used to create self-assembly systems.^198^ These intermolecular forces are dominated by hydrogen bonds, electrostatic interactions, hydrophobic interaction, and π–π interactions. Therefore, external stimulations to trigger self-assembly include the effect of pH, temperature, ionic charge and concentration as well as various other triggers such as enzymes and phototriggers. Different intramolecular driving forces and external stimulations can guide the self-assembly of polymer systems. These interactions have been extensively reviewed and are summarized in Table 3.^199−202^

**Table 3:** Driving Forces of Self-Assembly Adapted from Ref (205).

internal Interaction	strength (kJ/mol)	properties
electrostatic	50–300	electric force between charged bodies also known as Coulomb force; it can either be attractive between opposite charges or repulsive between like charges;^201^ short range interaction, nonselective
coordination binding	50–200	short ranged, directional
hydrogen bonding	5–120	interaction between hydrogen atoms and electronegative atoms; long ranged, selective, directional
π–π stacking	0–50	attractive noncovalent interaction between stacked aromatic rings; short ranged, directional
hydrophobic	depends on solvent type	hydrophobic segments are shielded from the aqueous solution by aggregating inside the self-assembled structure; this results from the van der Waals forces between hydrocarbon molecules and the hydrogen bonding between water molecules; affected by ionic strength.^206^
van der Waals	<5	attractive force, short ranged, nondirectional, nonselective
covalent	350	short ranged, irreversible

It is important to consider these known driving forces when tailoring the topographical, mechanical and electrical properties of SAPs for neural regeneration. Strong interactions such as ionic forces and coordination bonds require consideration in the design of a system that will self-assemble in the conditions found within the nervous system. Weaker interactions such as van der Waals electrostatic and hydrophobic interactions, H-bonding, and π–π stacking have strong influences on the self-assembled morphology, mechanical properties and bioactivity of SAPs. A balance between these forces can create molecules that will self-assemble into fibers in aqueous conditions but form a hydrogel when strong ions are introduced, thus enabling control over their gelation and subsequent material properties.^203,204^

To date, various types of self-assembling molecules in physiological environments have been explored ranging from synthetic small molecules, proteins, peptides, nucleic acids and hybrids as detailed in Table 4.

**Table 4:** Materials Used for Molecular Self-Assembly

hydrogelators	typical dominant forces driving self-assembly	features of the material	examples of neural cell response
DNA and nucleic acids^220^	base pairing–hydrogen bonding	can be tailored to incorporate specific molecular recognition and exhibit excellent biocompatibility; mostly researched for cancer applications^221,222^	DNA nanotubes covalently functionalized with RGDS epitopes; neural stem cells cultured on bioactive DNA nanotube substrates showed enhanced differentiation into neurons^215^
proteins and short peptides^198^	arrangement of hydrophobic and hydrophilic segments dictate secondary structure; hydrogen bonding, van der Waals electrostatic and hydrophobic interactions, H-bonding, and π–π stacking have all been used as gelator forces	can be protein functionalized with self-assembling short peptide sequences or short peptide sequences; low cost, biocompatible	RADA-16I showed axonal infiltration and strong integration with host tissue when injected after a spinal cord contusion lesion,^223^ and when seeded with HCMECs/D3 cells they promoted vascularization and augmented the host axon infiltration^224^
hybrid biomolecules^225^	electrostatic interactions between the charged AAs of the hydrophilic head, hydrogen bonding in the β-sheet forming regions as well as hydrophobic tail aggregation are dominant	typically consists of peptides segments functionalized with aromatic or alkyl groups; can also be DNA functionalized onto synthetic polymer chains self-assembly properties	Fmoc-FF: mulitpotent pericytes cultured for a week on the surface of Fmoc-FF/S coassembly showed neural differentiation on 1 kPa gel substrate^226^
	π–π stacking may occur if the synthetic component contains aromatic groups or the amino AAs contain aromatic groups in their side chains; can also rely on base pairing in ssDNA		peptide amphiphile: IKVAV functionalized self-assembling peptide amphiphile induced neural trans-differentiation in human bone marrow mesenchymal stem cells^227^
synthetic^228^	hydrophobicity, ionization and conformational change	block copolymers or designer small molecules that mimic self-assembly mechanisms found in nature	thermoresponsive PEG–PLAL loaded with BDNF and NGF led to neuronal differentiation of tonsil derived mesenchymal stem cells^210^

**Figure 3:**
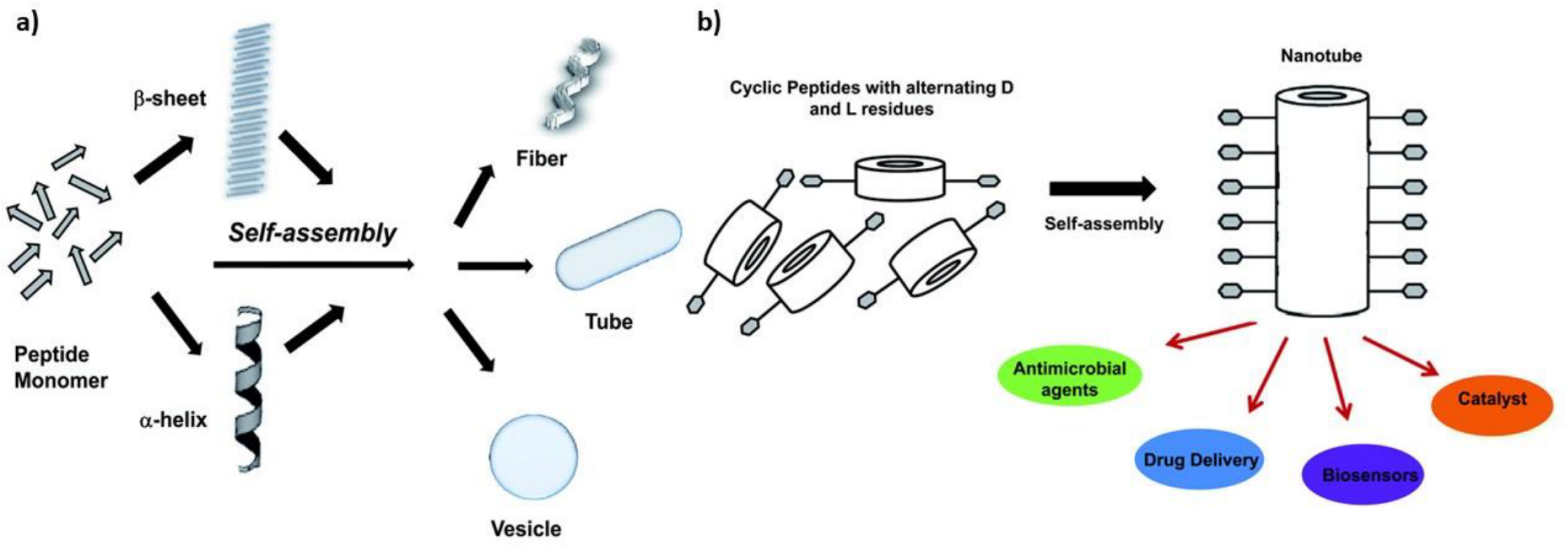
Possible self-assembled structures secondary and tertiary structures of (a) linear peptides and (b) cyclical peptides Adapted with permission from ref (212). Copyright 2010 Royal Society of Chemistry.

The chemical structure of these molecules allows control over size, shape, charge, and surface properties while maintaining low cytotoxicity.^207−209^ Most of these self-assembling molecules are in part driven by the interplay of hydrophobic and hydrophilic forces. For example, synthetic block copolymers comprised from alternating hydrophobic poly(l-alanine) and hydrophilic poly(ethylene glycol) segments form a self-assembling gel in aqueous conditions and this has been shown to support neuronal differentiation when loaded with growth factor releasing microspheres.^210^ Similarly, the RADA16-I is a peptide consisting of 16 AAs with alternating hydrophobic and hydrophilic residues. This drives its self-assembly in aqueous environments into a stable β-sheet structure.^19^ Alternately, Watson–Crick base pairing in DNA can be utilized to form self-assembling nanotubes of DNA segments, which can be functionalized with peptide sequences that promote neural differentiation.^211^ A common example of hybrid biomolecules are peptide amphiphiles (PAs), which consist of a hydrophilic peptide head, often followed by a β-sheet forming sequence, which is then capped with a hydrophobic segment. This leads to hydrophobic collapse in aqueous conditions.^200^ The hydrophobic tail can consist of alkyl chains, aromatic molecules such as Fmoc or other functional molecules.^212,213^ Nucleic acids and peptide or peptide amphiphiles are an ideal material because of their inherent low immunogenicity and versatile biofunctionality.^214−216,98,57^ PAs can easily be synthesized on both small scales for experimental study and large scale for application in the clinic.^217^ Peptides can also be functionalized with synthetic molecules in order to create amphiphilic molecules that self-assemble into a variety of different morphologies including fibers which promote the differentiation and elongation of neural stem cells, serving as a topographical guide for their growth.^218,219^ These self-assembling systems can be utilized to make materials across multiple length and spatial scales.^207^ Some of the most common morphologies are linear, trigonal, and cyclical structures, which then self-assemble to form various secondary and tertiary structures as illustrated by Figure 3.

### 3.1 Topographical Material Modifications

**Figure 4:**
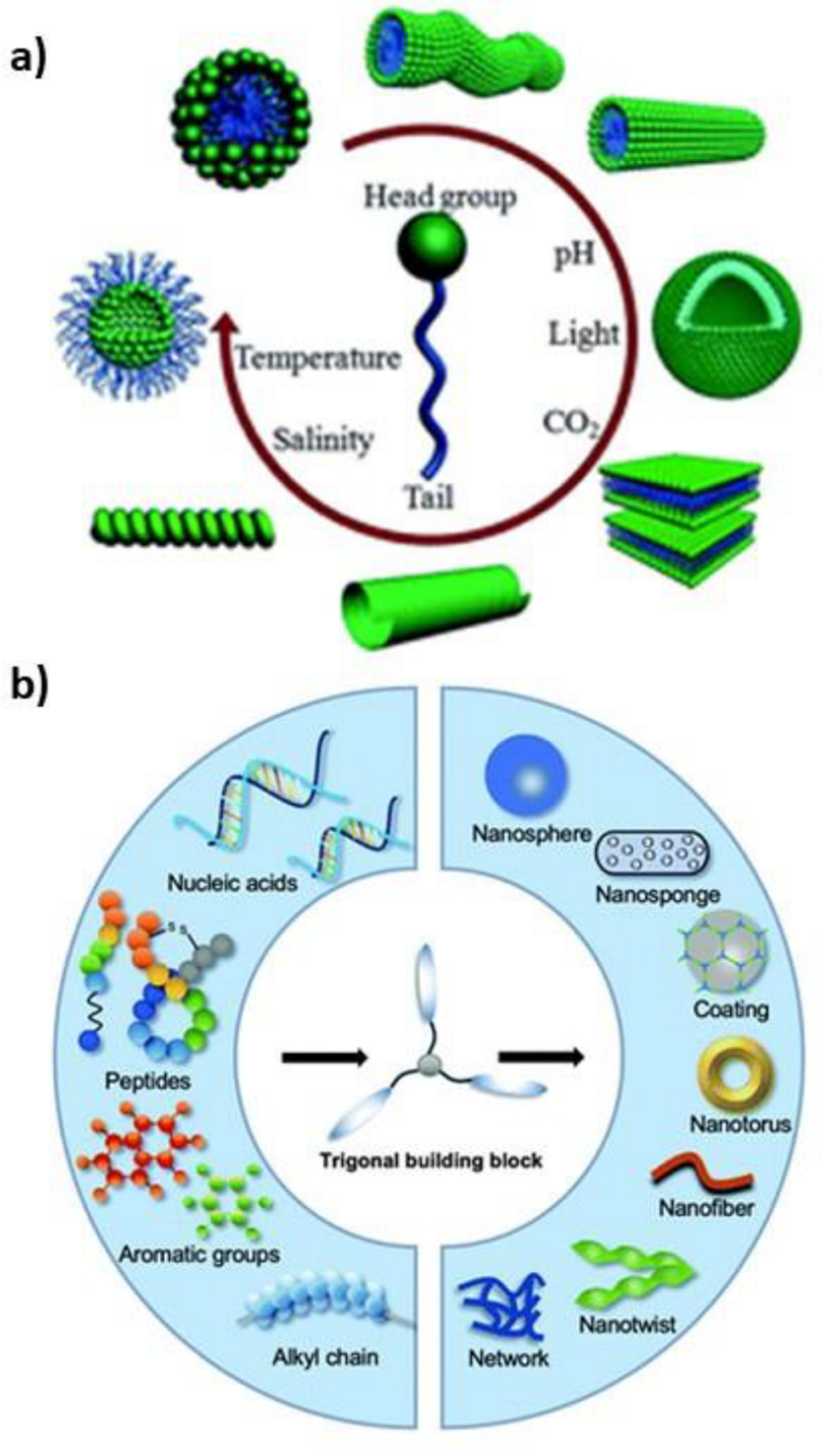
Schematic illustrations of self-assembled structures formed from various building blocks. (a) Amphiphilic building blocks adopting different morphologies. Reprinted with permission from ref (240). Copyright 2014 Royal Society of Chemistry. (b) Trigonal building blocks yielding different structures and morphologies. Reprinted with permission from ref (209). Copyright 2013 Royal Society of Chemistry.

**Figure 5:**
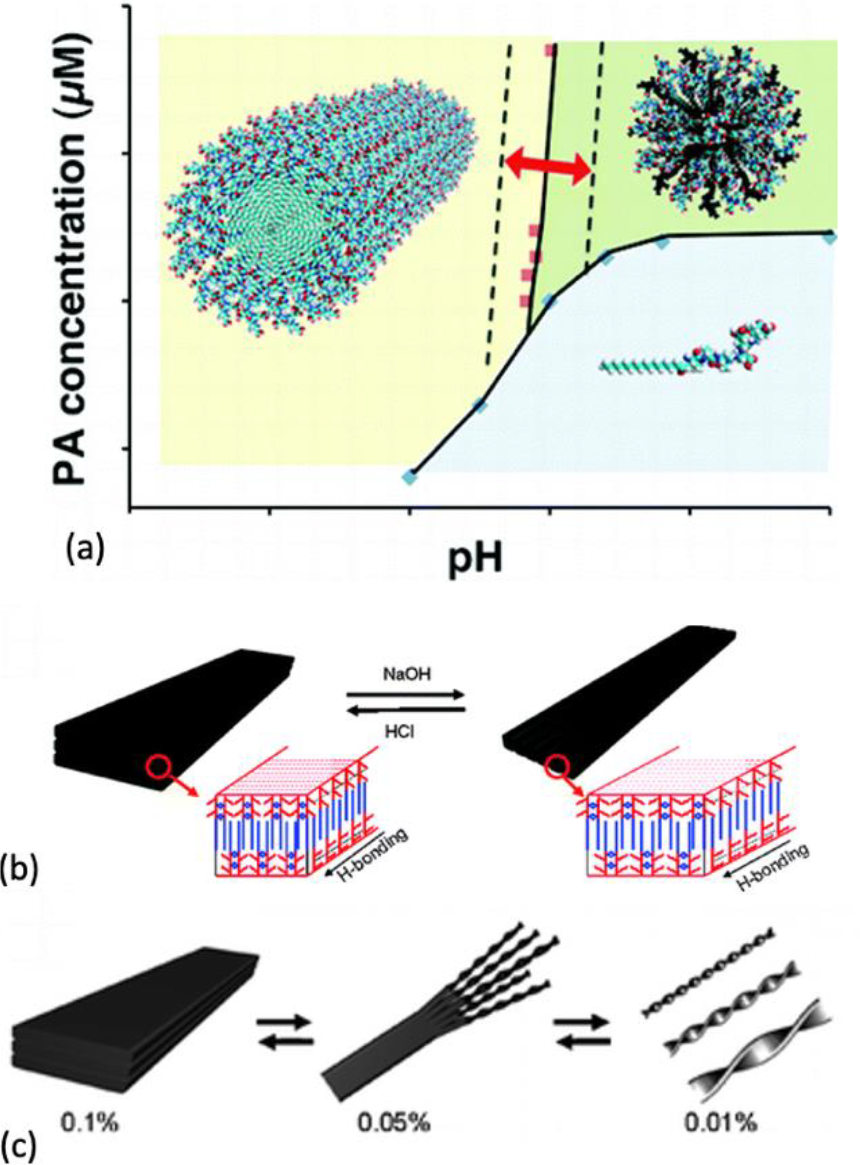
Effect of pH and concentration on self-assembly. (a) pH-dependent micellar, fibrillar, or dispersed topography. Reprinted with permission from ref (233). Copyright 2012 American Chemical Society. (b) Schematic illustration of pH change leading to the formation of nanobelts and varying concentration leading to a change in morphology from plaques to nanoribbons; (c) schematic illustration of morphology changes due to change in concentration. Reprinted with permission from ref (235). Copyright 2009 American Chemical Society.

The nanotopography of self-assembled structures can be modulated by varying the molecular structure or the environment in which the self-assembly occurs. More specifically, techniques such as changing the molecular design, electrostatic capping, pH, self-assembly molecule concentration or solvents have all been used to control the formation of micelles, β-sheets, α-helix, nanobelts, and membranes.^229−232^Figure 4 illustrates various structures formed under different conditions. For example, Ghosh et al.^233^ developed a PA that would transition from molecules dispersed in solution to micelles or nanofibers based on pH. A reduction in pH of 0.8 transformed micelles into nanofibers.^233^ This pH and concentration responsiveness is illustrated in Figure 5a. and can be used to design an injectable construct for neural repair which self-assembles when exposed to physiological pH.

The morphology of a self-assembly structure can also be fine-tuned by pH as shown by Cui et al.^234^ By varying the pH it was shown that a flat amphiphilic peptide nanobelt could be transformed into a grooved nanobelt with parallel nanochannels.^228^ Interestingly, a concentration-dependent modulation of morphologies was also demonstrated.^228^ Different structures including a split nanobelt with bristle morphology and twisted nanoribbons were achieved by reducing the concentration of PA molecules in the aqueous solution.^235^

Self-assembling fibers can be hierarchically organized in supramolecular crystals which can be aligned using various methods such as acoustic fields, pressure, magnetic fields,^236^ ultrasonication, electric fields, or external force fields.^214,237,238^ For example, Zhang et al.^239^ used shear force from the injection of an aqueous PA into an ionic solution to form a noodle-like hydrogel of aligned peptide nanofibers. This aligned PA was later functionalized with IKVAV and RGDS bioactive epitopes and shown to promote aligned neurite outgrowth in P19 mouse neurons.^218^ It also resulted in the formation of synapses and spontaneous electrical network formation after 2 weeks in culture with hippocampal neurons.^218^

Co-assembly is the incorporation of two or more distinct building blocks that self-assemble to form a structure, similar to the coassembly of proteins in nature. The combination of distinct components allows for the development of novel functional properties, and the tuning of supramolecular morphology and bioactivity as well as the physicochemical properties of the hydrogel. Various methods exist to obtain coassembly harnessing aromatic interactions, enzymatic action, electrostatic interaction, chemical stimuli, or electromechanical stimuli.^241^ Co-assembly and AA modification can also change dimensions and sizes of fibrous aggregates, fostering the formation of 1D or 3D networks.^242,243^ These techniques can be harnessed to create nanotopographies that can promote neural regeneration. Co-assembly can also be used to incorporate bioactive epitopes into the fibers in order to control cell fate.^244^

### 3.2 Mechanical Material Modifications

Self-assembling structures have tunable mechanical properties. By varying sequence charge, branching,^245^ concentration,^246,247^ coassembly, cross-linking,^248^ and solvent/ions interactions the mechanical properties can be tailored.^212,249−251,213,252^ The mechanical properties of SAPs that have been achieved using these methods can be found in Table 5. For example, Clarke et al.^249^ showed that by modifying peptide concentration and sequence charge of an oligopeptide the elastic modulus of the resulting hydrogel can be varied across 2 orders of magnitude from 2–200 kPa. Shear thinning and self-healing properties were also demonstrated through reassembly, which are of interest for in situ placement.^249^ Shantanu et al.^111^ explored the effect of varying gel stiffness on hippocampal cells. By varying the strength of the β-sheet interactions PAs with stiffness of 22.9 and 7.3 kPa were designed.^111^ Hippocampal neurons were subsequently cultured on peptide coated surfaces and it was found that the stiffness of the substrate greatly affected astrocyte density and neuronal maturation.^111^ Stiffer substrates led to an astrocyte density 10 times higher than softer substrates, while neuronal density was 30% lower on stiffer substrates compared to soft self-assembled fibers.^111^ This demonstrated that varying stiffness allows for control over the differentiation of neural cells.^111^ Furthermore, the effect of stiffness on neuron maturation, classified by morphological criteria, was apparent after only 20 h in culture.^111^ Interestingly, softer peptide amphiphile scaffolds showed faster maturation of neurons, which was not dependent on the presence of KDI or RGDS epitopes.^111^

**Table 5:** Stiffness of Various Self-Assembled Hydrogels in Cell Culture Conditions

method used to modulate stiffness	material	range of storage modulus (stiffness) obtained (kPa)	ref
concentration	RADA I	0.046–0.735	(246)
	RADA II		
concentration and sequence	2–15 mg/mL	0.5–3	(247)
	KFE-8		
	KFE RGD		
	KFE RDG		
	pentapeptide	2–200	(253)
co-assembly and concentration	SA5N	10–200	(255)
	SA21		
	Fmoc peptides	2–30	(256)
sequence modifications	peptide amphiphile	7–23	(111)
	branched (LDLK)3 peptides	0.002–0.008	(257)
cross-linking	self-assembled peptides cross-linked with genipin	1.5–120	(248)

Scaffold degradation allows cells to remodel the ECM, thus improving migration and viability.^118^ Degradation of self-assembling materials can be tuned by varying the molecular structure. For example, the incorporation of sequences that can be cleaved by MMPs has led to the degradation of β-sheet fibrillar materials.^125,36,258^ However, the expression of MMPs is hard to control in vivo. An alternative that has been investigated is the incorporation of ester bonds into self-assembled gels, rendering the degradation dependent on pH and water accessibility, a more predictable in vivo process. Collier et al.^259^ showed that by incorporating glycolic acid (Glc) within the peptide segment of an Fmoc-F-RGD SAP resulted in a linear degradation profile over 60 days. Placement of the Glc segment was critical, as substituting the glycine in the RGD sequence resulted in greatly reduced bioactivity of the adhesion epitope.^259^ Placement of the Glc segment next to intact RGD sequences permitted hydrolytic degradation without compromising the bioactivity of the RGD sequence.^259^ The stiffness of the degradable gel was around 1.5 kPa.^259^ When coassembled with and Fmoc-diphenylaniline peptide which has a stiffness of 30 kPa, a range of stiffnesses was obtained depending on the ratio up to a stiffness of 13 kPa for a 20:1 ratio of Fmoc-FF to Fmoc-F-Glc-RGD.^259^ Rho et al.^260^ showed that secondary hydrophobic interaction near the core of cyclical peptides can stabilize the peptide bonds without compromising on solubility in aqueous conditions.

### 3.3 Incorporating Biomolecular Components

A wide range of bioactive cues have been incorporated within biomaterials intended for neural repair. SAPs offer the possibility of multifunctionalizing the material system, by simultaneously incorporating bioactive molecules in the peptide sequence and within the scaffold structure. The versatility of their biofunctionalization is a major advantage in the field of neural scaffold materials.^152,153^ An overview of recently explored bioactive cues incorporated in SAP materials is presented in Table 6. Adhesion molecules consist of bioactive epitopes derived from large molecules found in the neural ECM and they interact with the cells through integrins.^138^ These molecules are necessary for cell survival, migration, and differentiation and cell behavior can be influenced by modifying the scaffold’s adhesion cues.^261^ Decellularized ECM materials or purified single ECM components can be engineered as injectable natural scaffolds to preserve the physiological chemical environment.^42,65,138,262^ Hyaluronan, methylcellulose, chitosan, and fibrin among other materials can be used to design in situ forming biomaterials for neural repair and drug delivery.^42,65,263,264^ However, such materials can present batch-to-batch variability, and tuning their composition or material properties can be challenging.^39,42^ Synthetic SAPs offer the possibility of multiple functionalizations with targeted molecules and epitopes in predefined concentrations.^130^ Thus, bioactive ECM components can be included in self-assembling material design maintaining constant biochemical and physical conditions.^58,265^

**Table 6:** Bioactive Molecules for Neural Engineering Incorporated in SAPs

bioactive molecule	SAP system	inclusion method	physical and biological action	ref

Adhesion Molecules				
IKVAV	RADA16-IKVAV	addition at one extremity of the peptide sequence by covalent bond	enhanced survival of encapsulated NSCs and glial scar reduction	(134,268)
			improvement of neuroregeneration after 6 weeks in traumatic brain injury murine models	
	RADA16-IKVAV/-RGD	addition at one extremity of the peptide sequence by covalent bond and SAP combination	increased spinal cord embryonic primary cell viability and increased neural differentiation compared to 2D substrates and nonfunctionalized peptide (RADA16-I)	(296,297)
			enhanced neural differentiation in primary embryonic rat NSCs in vitro	
			promoted nerve functional regrowth in vivo	
	PA-IKVAV	addition at one extremity of the peptide sequence by covalent bond	increase in neural stem cell neurogenesis and neuronal differentiation in vitro	(267,275,166)
			improvement in Alzheimer’s symptoms and neurogenesis in vivo	
YIGSR	RADA16-GG-YIGSR	addition at one extremity of the peptide sequence by covalent bond	increase in neuronal differentiation, restoration of memory/learning function in Alzheimer’s mice models, rescued synaptic function, decrease in pro-inflammatory factors	(234)
RGD	RADA16-I	addition at one extremity of the peptide sequence by covalent bond	promoted primary murine NSC proliferation and differentiation with mechanical and rheological properties comparable with neural tissue	(272)
	RADA4	addition at one extremity of the peptide sequence by covalent bond	supported proliferation and differentiation of primary mouse NSCs compared to Matrigel control	(298)

Growth Factors				
BDNF and GDNF	Fmoc-DDIKVAV	SAP functonalied with chitosan molecule, cross-linking between chitosan polysaccharide amine group and BDNF sulfhydryl group	increase GF lifespan by over 40 times	(101)
			structural and biochemical peptide properties maintained	
βFGF	RADA16-DGE	electrostatic interaction with negatively charged peptide terminus	clinically viable drug release profiles, increased neural stem cell proliferation	(283)
GDNF	Fmoc-DIKVAV	addition of IKVAV at one extremity of the peptide sequence by covalent bond	sustained released of GDNF from 1 to 172 h	(282)
		GDNF physical entrapment by gelation	implants of cell-loaded material system in Parkinson’s disease murine models promotes graft cell survival, reinnervation of the host tissue, and overall endogenous tissue repair	
bone marrow homing peptide 1 and 2 (BMHP1, BMHP2) motifs	RAD16-I	GF motives directly extended from the peptide sequence by covalent bond	enhanced NSC survival and differentiation promoted differentiation toward neural and glial fate in vitro	(286)
			amelioration of locomotor recovery in rats	

Drugs and Proteins				
lipophilic drugs (pindolol, quinine, and timolol maleate)	RADA16-II	physical entrapment	clinically viable release profile of lipophilic drugs was obtained, while maintaining the peptide nanostructure	(299)

**Figure 6:**
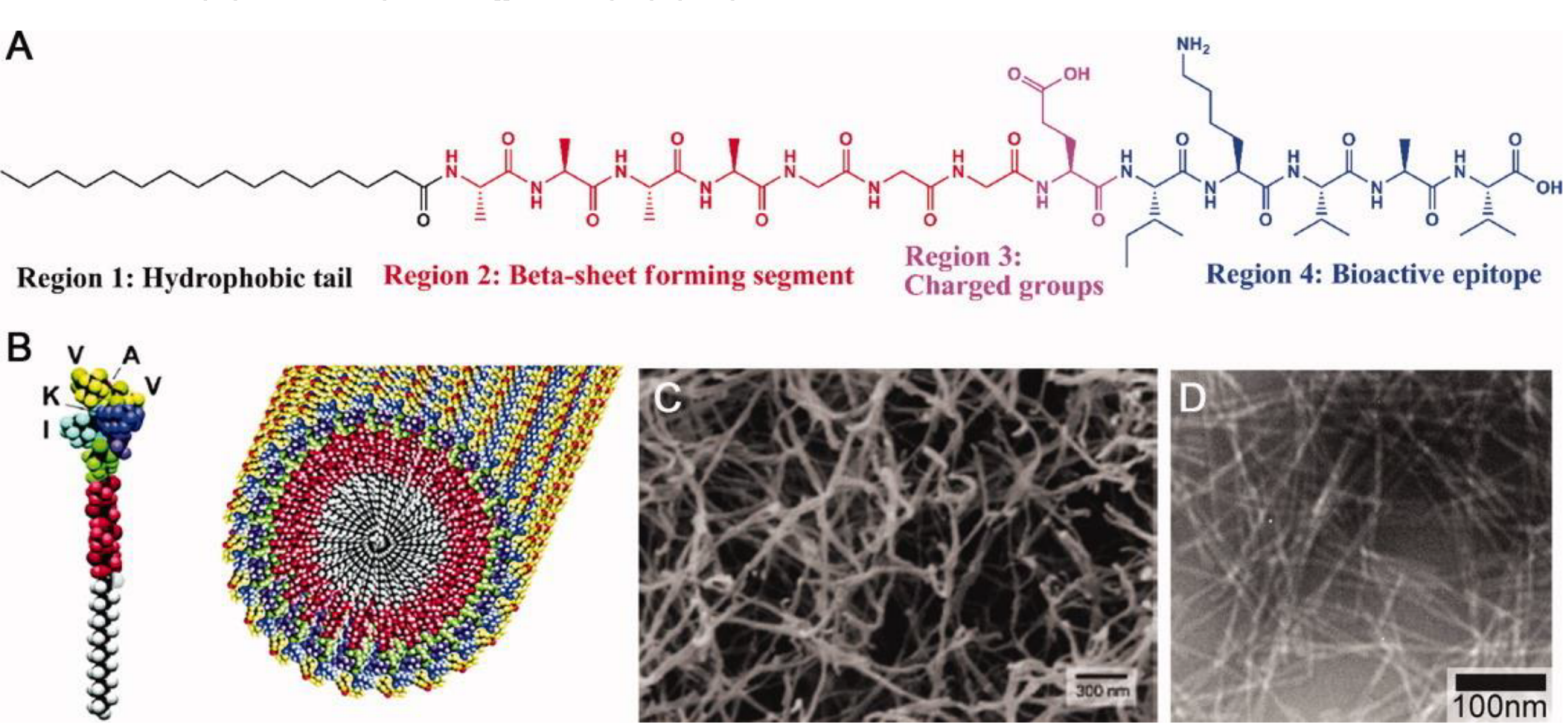
SAP PA-IKVAV. (A) Molecular structure composed of four functional regions dedicated to different functions, highlighting the versatility and multifunctionality of SAP systems. (B) Molecular graphics of the PA-IKVAV molecules, also assembled into a nanofiber. (C, D) Scanning electron microscopy and transmission electron microscopy (respectively) of self-assembled PA-IKVAV nanofibers. Reproduced with permission from refs (276) and (273). Copyright 2004 The American Association for the Advancement of Science and 2010 John Wiley and Sons.

Adhesion epitopes can be introduced in the peptide sequence, and therefore they are often designed to be as short as possible so as not to interfere with the nanostructure and self-assembly mechanism.^58^ Neural bioactive peptide motifs tested for use in biomaterials are usually derived from the amino-acidic sequence of the neural cell adhesion molecule (NCAM), fibrin, laminin, and fibronectin.^34,58^ Aside from the universal adhesion molecule RGD laminin-derived epitope, IKVAV can be considered the most popular example in neural engineering for its role in neural stem cell differentiation and glial scar reduction, especially when combined with SAPs such as RADA-IKVAV and PA sequences.^97,266−268^ Epitope peptides can be synthesized directly at any site of the SAP backbone sequence or chemically ligated as a postsynthesis modification, in a linear or branched fashion.^265,269−272^ Solid phase peptide synthesis is one of the most common techniques, chosen for the relatively simple method and versatility.^270,273−275^ Investigation on the effect of different bioactive epitopes, epitope density and exposure are possible because of the highly controllable chemical structure of SAPs and precise material purification methods.^97,275^ Silva et al.^275^ synthesized a peptide-amphiphile material that assembles into nanofibers at physiological pH and functionalized it by chemically binding the IKVAV epitope at one extremity of the sequence (Figure 6).^276^ The PA-IKVAV showed optimal NSC survival compared to 2D laminin controls in vitro.^275^ The IKVAV epitope density was then modified by mixing the material with different concentrations of the same SAP sequence functionalized with a nonphysiological sequence instead of IKVAV.^275^ The results showed that neuronal differentiation increased with IKVAV epitope density as opposed to astrocytic development.^275^ The same material was shown by Yang et al.^267^ to improve cognitive impairments and increase hippocampal neurogenesis when implanted in Alzheimer’s transgenic mice. Tysseling-Mattiace et al.^267,277^ also reported the reduction glial scar formation, the regeneration of sensory fibers and significant behavioral improvements in an in vivo murine model of spinal cord injury. Cui et al.^234^ presented similar results with the SAP RADA16 functionalized with the motif YIGSR, a laminin-derived epitope that also promotes neural differentiation and proliferation. These results demonstrate the effectiveness and versatility of SAPs in disease-targeted neuroregeneration.

Combining multiple functionalizations within the same SAP can be used to target different pathways and achieve synergistic effects. For example, Galler et al.^278^ synthesized a multidomain SAP containing both the degradable MMP-2 motif and the adhesion peptide RGD in different peptide locations, observing enhanced cell viability, spreading, and migration. The epitope distribution and topography can also be controlled through chemical interactions with specific AAs,^34,279^ thereby affecting the cell overall behavior. Sur et al.^279^ functionalized PA nanofibers by binding RGD epitopes on specific glycine sites, which was shown to affect cell spreading on the scaffold nanostructure.

**Figure 7:**
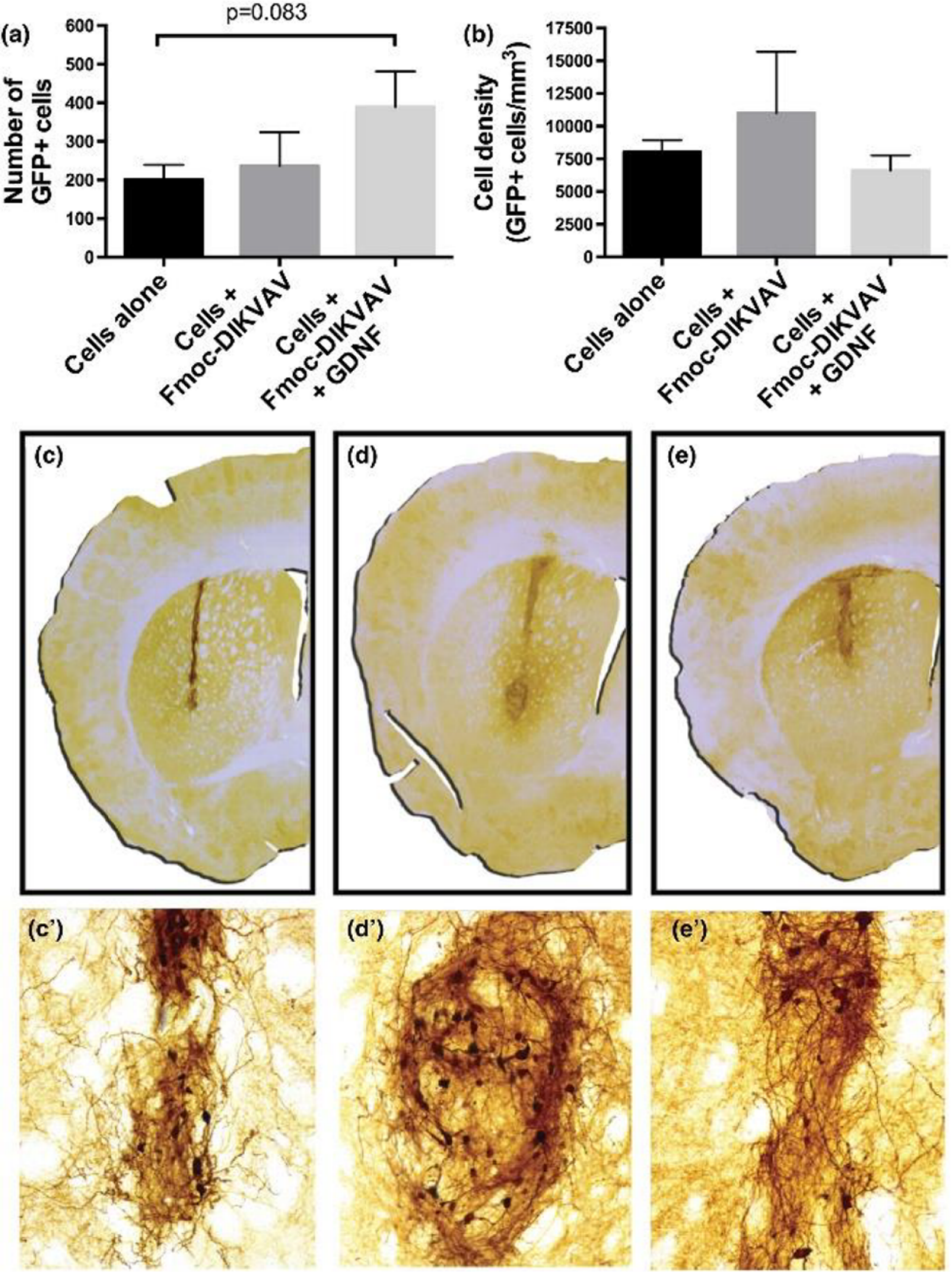
In vivo effect of a SAP biofunctionalized with the adhesive molecule IKVAV and the growth factor GDNF in a Parkinson’s disease murine model. (a, b) The effect of the functionalized hydrogel is more pronounced than the cell implanted alone, as shown by the GFP+ cell density 10 weeks post-transplantation. The transplant has different outcomes in vivo, where (c) the cell line alone showed a lower graft survival than (d) the cells with the SAP N-fluorenylmethyloxycarbonyl (Fmoc)-DIKVAV and (e) the SAP combined with the GDNF growth factor. Reproduced with permission from ref (282). Copyright 2017 John Wiley and Sons.

In addition to adhesion molecules, other bioactive elements such as GFs and neurotrophins can affect both cell behavior and cell fate.^130,280^ GFs are a powerful and widespread tool for regeneration applications, however their administration route and method must be finely controlled because of the short half-life, relatively large size, slow tissue penetration, and the potential toxic effects at high levels when delivered systemically.^280^ SAPs are considered an optimal GF delivery method because they offer protection from degradation, controlled spatial and temporal release and local administration.^280−282^ GF molecules can be inserted directly into the SAP sequence as seen for adhesion molecules,^281,283^ or they can be chemically conjugated with the hydrogel. Other methods of incorporation include the use of GF-specific binding sequences or biotin–streptavidin–biotin bonds.^43,281,284,285^ Gelain et al.^286^ extended the peptide sequence of RAD16-I by directly adding bone marrow homing peptide 1 and 2 (BMHP1, BMHP2) motifs, achieving an increase in primary NSC proliferation and neural differentiation, whereas maintaining a stable and precise GF delivery and concentration. GFs can also be encapsulated into the polymer network through physical bonds which break upon hydrogel degradation.^43,282,283^ Finally, Gelain et al.^283^ achieved clinically viable GF release profiles incorporating negatively charged AA sequences to the SAP RADA16-I. The positively charged basic-fibroblast cytokine (βFGF) electrostatically interacted with the SAP terminus, allowing for a gradual release, which increased NSC proliferation.^283^ GFs can also be combined with adhesion epitopes using different incorporation methods, to enable effective delivery to the tissues. Rodriguez et al.^282^ synthesized the SAP Fmoc-DIKVAV as a single peptide chain and subsequently incorporated glial cell line derived neurotrophic factor (GDNF) by physically entrapping the molecule within the hydrogel upon gelation. This allowed for a dual effect on NSC differentiation and proliferation by the IKVAV epitope and NGF, which improved the regeneration effect of a NSC transplant in Parkinson’s disease mice models (Figure 7)^282^

Self-assembling drug-loaded microparticles^287,288^ and genetically modified cells for GF production are other delivery approaches.^289^ RAD16-I was employed to create cell-encapsulating microgel beads, which were able to support cell proliferation and diffusion of nutrients.^130^ Indeed, other signaling proteins and drugs such as neurotransmitters, gene vectors, and signaling molecules can be encapsulated into the SAP structure for self-assembling, resulting in delivery profiles similar to GF delivery.^43,56,283,290−293^ The MAX8 β-hairpin SAP designed by Branco et al.^290^ exploits the positive charge of the hydrogel network to bind and release negatively charged molecules with different isoelectric points. Koutsopoulos et al.^291^ investigated the delivery properties of RADA16 with different proteins physically encapsulated during the self-assembling process. The findings reveal the structural stability of the SAP when employed as a drug delivery system, and the size-dependent protein release.^291^ Importantly, the molecular structure, size, charge, and biological effects need to be investigated case by case to reach an appropriate release and administration route.^290,291^

SAP material systems can also be used to design stimuli-controlled drug delivery systems.^43^ Different physiological stimuli can modify the material interaction with the encapsulated bioactive molecule and trigger its release.^43^ For example, the material degradation of a Fmoc-based SAP can be tuned with the temporal release of GF motifs, resulting in optimal drug release profiles, as shown by Bruggeman et al.^101^ Drugs and molecules can also be linked to the material with enzymatically cleavable bonds,^125,278,294^ or chemical links subject to change in pH, temperature, and magnetic fields.^43,56,135,295^ This feature introduces significant advantages for delivery approaches that require spatially or temporally targeted delivery methods.

## 4 Considerations for Electrical Stimulation

Electrical stimulation is a powerful tool for neural repair. The therapeutic effect of stimulation is supported by a range of treatments targeting diverse injury settings and applications.^14,300^ It is therefore important to consider how electrical stimulation can be incorporated in self-assembling hydrogel systems in order to achieve neural regeneration. Examples of widespread clinically implemented electrical stimulation methods are deep brain stimulation (DBS) for brain diseases and functional electrical stimulation (FES) for spinal cord injuries.^14,301^ However, although these devices are designed to replace lost function, without scaffold support, there is minimal capacity for neural tissue regeneration. In fact, the implantation and presence of a rigid device can result in further neural cell loss. The employment of scaffold materials within bioelectronics applications has been gaining attention over the past decade, including the use of soft polymeric electronics for implants, neural interface coating materials, and drug delivery systems.^302,303^ These technologies have revealed both the potential for organic conductors applied in electrical stimulation and the need for scaffold materials that are compatible with electrical stimulation.^30,304,305^ Coupling bionic devices with tissue engineered scaffolds is an emergent area where conductive SAPs may find application. However, it is essential to ensure the compatibility of the self-assembling mechanism, which is often driven by electrostatic interactions with the propagation of electrical signals.^191,196^

Electroactive scaffolds have been developed to actively promote electrical stimulation^116,306−310^ and ionically porous materials have been used to ensure that cells are effectively exposed to electric fields.^189^ Incorporation of conductive materials into self-assembling scaffolds has been investigated as a method of providing cell scaffolds with conductive elements. Carbon based nanomaterials such as nanotubes (CNT) and graphene have been explored to confer electroactivity to scaffolds. Although they demonstrate good conductivity and polymer composites have been designed with appropriate mechanical properties, the regulatory pathway for new materials and in particular carbon nanomaterials has hindered their clinical translation.^311,312^ Conductive polymers (CPs)^313^ have also emerged as a potential solution due to their high charge injection capacity and ionic conductance.^304,313−316^ CPs are characterized by alternating single and double bonds along the backbone, termed π-conjugation, which cause a delocalization of electrons. Some of the most commonly used CPs for in vivo studies are poly(3,4-ethylenedioxythiophene) (PEDOT), polypyrrole (PPy), and polyaniline (PANI).^60^ The primary disadvantage of CPs are their poor mechanical stability and limited conformational control.^305,317,318^ The addition of bioactive cues to CPs to improve cell attachment and proliferation can also have a significant impact on the polymer properties, preventing the possibility of a multifunctional biomimetic scaffold from a bulk CP.^319^

To improve the scaffold electrical properties while maintaining cytocompatibility and mechanical tuneability, researchers can introduce conducting elements such as CPs or CNTs to softer and more tunable materials.^320^ Conductive hydrogels (CHs) have been developed pursuing this concept and applied to flexible bioelectronic applications.^314,321,322^ The coupling of these conductive materials to self-assembling hydrogels has also been investigated.^33,306,323−325^ Relevant examples of natural and synthetic self-assembling materials compatible with electrical stimulation or that possess intrinsic conductive properties can be found in Table 7. Peptides inspired by bacterial pili have shown some extremely high conductivities as reviewed by Hochbaum et al.^326^ However, this conductivity has been shown to be highly dependent on the secondary and tertiary structures,^327^ making it difficult to tailor these systems for neural repair applications.

**Table 7:** Conductive Self-Assembling Hydrogels and Polymers

material	electrical properties	degradation and toxicity	application	ref
tetra(aniline)-based cationic amphiphile self-assembled into a nanowire thin film	acid-doped emeraldine salt of aniline was spin-coated into a thin film and dried under a vacuum; conductivity of 2.7 ± 0.3 mS cm^–1^ was calculated based on four-point probe resistance measurements	self-assembled in aqueous solution but biocompatibility still needs to be studied	designed for application in sensors and device; gel formation was not investigated	(358)
amphiphilic peptide-functionalized with an alkyl spacer and tetra(aniline)	tetra(aniline) fibers were doped with HCl and dried under a vacuum; conductivity was measured via a two-point probe to be 6.97 × 10^–6^ S/cm	co-assembled into a porous nanofiber network with a diameter of 10 nm; PC12 study showed good biocompatibility	PC12 study showed improved neurite outgrowth and more advanced differentiation after 6 days in vitro; demonstrated the potential as an electroactive scaffold for neural culture in vitro	(357)
tetra(aniline) terpolymer that forms aggregates with a TANI core and PEG corona	drop-cast aqueous samples had a conductivity of 2.1 × 10^–4^ when measured via a four-point probe	coculture with chondrocytes showed good biocompatibility and the gels showed a 90% decrease in viscosity over 100 min in PBS at 37 °C; in vivo systemic injection showed strong electroactive intrinsic antioxidant behavior	showed potential for treatment of oxidative stress in diabetic rats yielding normalized ROS levels and enzymatic antioxidants	
amphiphilic peptide functionalized with an alkyl spacer and BTBT	displayed extended π-delocalization within the hydrophobic core resulting in a conductivity of 6.0 × 10^–6^ S cm^–1^ without doping	self-assembled into nanofibers of 11–13 nm in aqueous media but no cell studies have been made	bioelectronics and possibly tissue engineering	(359)
single-walled carbon nanotubes in collagen and Matrigel hydrogel	bulk conductivity of 1723 ×10^–3^ S/m	improved neurite outgrowth dorsal root ganglia primary cells from P2 neonatal rats under 8 h DC stimulation	elastic modulus of 37–50 Pa	(306)
bundled carbon nanotubes entrapped in β-Vhex nanofibers	conductivity of 0.02 S/cm and impedance of 0.2 MΩ as measured by filling a nonconductive microtube with gel and placing 2 electrodes on each side	biostable with little degradation when injected into the brain cortex; no difference in microglial activation relative to the control	soft neural interface to improve neural signal recordings	(360)
PEDOT polymer confined within peptide amphiphile nanostructures	finite window of conductivity: maximum on the forward sweep at 5.52 × 10^–5^ S cm^–1^ at 0.12 V and global maximum of 6.57 × 10^–5^ S cm^–1^ on the reverse sweep at −0.01 V			(33)
chitosan/gelatin porous scaffolds assembled with conductive poly(3,4-ethylenedioxythiophene) nanoparticles	5.82 × 10^–5^ to 6.22 × 10^–1^ hydrated, acellularized, 6.45 × 10^–5^ to 6.81 × 10^–1^ with cells	30–70% biodegradation in 8 weeks, PC12 cell viability maintained throughout the study	in vitro study of PC12 cell viability, adhesion and proliferation, morphology and epigenetic investigation	(361)
Fmoc-FF-PANI hydrogel	from 10 to 2 to 10^–1^ S/cm; high cell viability of cardiomyocytes grown on the composite hydrogel demonstrates its noncytotoxicity	degradation of ∼62% was obtained after 20 days	living dynamic range pressure sensing and electroconductive interface for electrogenic cardiac cells	(362)
conductive collagen/poly pyrrole-*b*-polycaprolactone hydrogel	1–5 mS/cm	PC12 cell viability did not differ from collagen after 48 h of incubation	bioprinting for neural tissue constructs	(363)
bacterial derived α-helix peptide self-assembly into nanofibers	shows ohmic charge conduction in aqueous states and conductance AFM measured a conductivity of 1.12 ± 0.77 S cm^–1^ for individual nanofibers; conductance of 10^–6^ S cm^–1^ for a 0.3 wt % gel measured with EIS; interestingly, conductivity decreases with increasing peptide concentration		potential for bioelectronics applications	(327)
peptide thiophene hybrids	1wv% peptide hybrid gels combined with EDOT–OH and pTSA; conductivities range from 3 × 10^–3^ to 1.5 × 10^–2^ S cm^–1^	storage modulus ranging from 27 to 100 kPa, no cell studies	possible application in tissue engineering	(364)
polydiacetylene conjugated peptide consisting of a polymerized polydiacetylene core flanked by peptides			self-assembled hydrogel of aligned nanofibers with polymerized polydiacetylene at the core	(365)
diphenylalanine peptide nanowires		2D culture of primary neocortical neurons exhibit enhanced viability, neurotransmitter release, and lower fraction of non- oxidative glucose metabolism	2D cell culture	(366)
T4P-like peptide decorated with gold nanoparticle	445–427 S cm^–1^	cardiac cell cultures on nanofiber film for 5 days; the film supported the assembly of single cells into synchronized cardiac patches	microelectronics, sensors, integrated in electroresponsive tissues	(367)

### 4.1 Conductive SAPs

Self-assembly of small molecules enables a bottom-up control of material properties. Aromatic compounds are mostly incorporated into hydrogels for biomedical applications because they enhance the formation and stability of hydrogels in self-assembling systems.^328^ For example, Fmoc functionalization of SAPs have been shown to aid self-assembly by enhancing π–π stacking.^329^ In peptide-based hydrogels, aromatic compounds are used as gelators, helping form hydrogels that are mechanically stable and biocompatible for various applications such as drug carriers or antifouling/antibacterial gels.^330^ Aromatic compounds are used to cap the N-terminus in solid phase peptide synthesis, and can therefore easily be integrated into the material synthesis.^331,328^ The incorporation of aromatic compounds and oligomers in self-assembling molecules has been explored to make conductive materials for a wide variety of applications such as electronics, optics, optoelectronics, photovoltaics, magnetic and piezoelectric devices, sensors, drug releasing hydrogels, and catalysts.^328,330,332−339^

The field of nanoarchitectonics has studied the hierarchical organization of self-assembling molecules into functional layers, sensors, bioactive components, and artificial living systems. Conductive layers have therefore been developed by incorporating aromatic molecules and small linker molecules to various self-assembling molecules.^340^ Supramolecular electronics have studied the assembly of π-conjugates into electronic nanowires.^341^ Many of these self-assemblies occur in organic solvents, which inherently limits their application to neural regeneration where a key design criterion is *in situ* formation via injectable preproducts. Furthermore, the stability and degradation of electroactive scaffolds in physiological environments is not well understood. Despite these current limitations, the approaches taken in these associated fields that employ self-assembly techniques demonstrate the tuneability and feasibility of developing 3D conductive self-assembling networks for neural repair.^342,343^ For example, highly conductive BTBT amphiphiles are commonly formed in organic solvents, but recently, it has been demonstrated that self-assembly of these molecules can be achieved in aqueous conditions, in a first step toward a tissue engineering application.^344^ Interest in these π-conjugated peptides for biomaterial applications will continue to grow because of the tuneability and nontoxic nature of self-assembling electroactive molecules.^345,346^ Understanding the various methods that have been used to tailor the electronic properties of π-conjugated oligomer self-assembling systems is crucial to determining their compatibility for neural tissue engineering.

To develop a biomimetic scaffold understanding how the incorporation of π-conjugated systems affects topographical, mechanical, and conductive properties is key. Varying the molecular structure has been shown to tune the secondary structure and morphology of aromatic self-assembled molecules.^347−349^ Peptide π-conjugates are often composed of an AA region, a CP region, and sometimes a hydrophobic alkyl tail region. Modifying the various components of this molecular structure can affect several material properties. Lehrman et al.^350^ showed that by varying the AA side chain of peptide thiophene structures allows for control over the resulting nanostructure. By substituting AA of varying size and hydrophobicity, it was demonstrated that π–π stacking and hydrogen bonding both contribute to self-assembly but are also competitive forces.^350^ It was suggested that the control of nanostructures arises from the optimization of the balance between π–π stacking, intermolecular hydrogen bonding, and attractive van de Waals forces.^350^ Changing this balance results in different morphologies including flat structures, spiral sheets, or nanotubes.^350^ Panda et al.^351^ also showed that by varying the alkyl spacer length between the peptide and the aromatic component of the self-assembling structure the chirality of a triblock β-sheet fiber could be tuned. All-atom molecular simulations were subsequently used to design peptide sequences to control peptide chirality and electron delocalization properties.^352^ Peptide chirality affects the conformation and morphology of the resulting structure and is therefore of great interest for bioactivity.^353^ The core oligomer length was also shown to influence the phase behavior and morphology of self-assembled structures. Different oligomer lengths can lead to the formation of high-aspect-ratio fiber networks or disordered aggregates.^354^ Alternatively, varying the solvent has been shown to change the self-assembled morphology.^355,356^ Doping of the conjugated structures has also been shown to alter the morphology of self-assembled molecules. Mushtaq et al.^335^ showed that PEGylated tetra (aniline) self-assembled into spherical nanostructures. It was shown that these nanostructures can be doped using HCl, which increases their size.^335^ These structures were found to be electroactive through cyclic voltammetry and UV–vis spectroscopy investigations.^335^ These polymers were shown to have excellent cytocompatibility when injected into the liver of rats.^336^

Finally, it has been shown that biological benefits can be achieved from these electrically modified SAPs. Guler et al.^357^ incorporated tetra(aniline) into an SAP nanofiber and demonstrated that neurite outgrowth and differentiation of PC12 cells was enhanced 6 days after induction with NGF. It was demonstrated that this conductive SAP upregulated the phosphorylation level of the ERK1/2 pathway relative to the nonfunctionalized peptide in an investigation of the upstream pathways of NGF induced neural-like differentiation.^357^ Although these studies show the significant promise of SAPs, many do not characterize the SAP electrical properties in physiological conditions.

**Figure 8:**
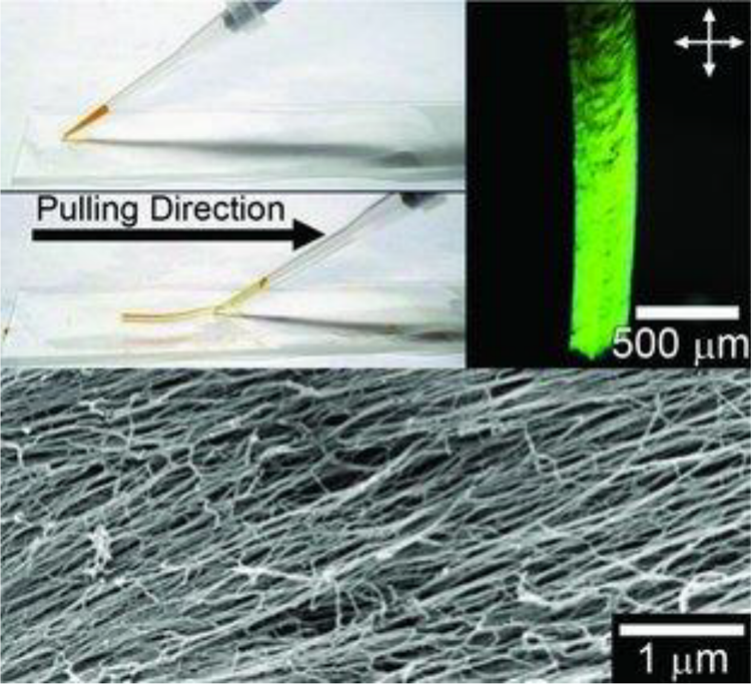
Alignment of π-conjugated peptide hydrogel using shear flow assembly. Reproduced with permission from ref (368). Copyright Advanced Materials 2011.

The hierarchical organization of self-assembling π-conjugated fibers is of interest for neural tissue engineering since combining their conductive properties with topographical cues could lead to synergistic effects on neural cells. Aligned fibers are of interest for both spinal cord and peripheral nerve regeneration as they mimic the topography found in vivo. Wall et al.^368^ demonstrated the hierarchical organization of self-assembling π-conjugated fibers into aligned macroscopic domains. Aligned macroscopic domains of optoelectronic structures were fabricated using a shear-flow assembly as shown in Figure 8.^368^ As discussed above, topographical cues from self-assembled aligned fibrous networks have been shown to guide neurite outgrowth, which is particularly applicable to mimicking spinal cord architecture for regeneration after SCI. Wu et al.^369^ studied the effect of combining topographical cues with the photoconductive polymer poly(3-hexylthiophene) (P3HT) on neurite outgrowth. The growth and differentiation of PC12 cells was assessed on a homogeneous P3HT film, self-assembled P3HT nanofibers, and electrospun P3HT/poly(e-caprolactone) (PCL) microfibers with and without light irradiation.^369^ This enabled an understanding of the effect of electrical stimulation due to the photoconductive polymer in conjunction with different topographies.^369^ The electrical stimulation provided by the LED irradiation was shown to consistently promote increased neurite outgrowth on all topographies and the self-assembled nanofibers promoted the longest neurite outgrowth.^369^

It is important to understand how these modifications to molecular structure will affect the conductivity of the self-assembled π-conjugates. Ardoña et al.^370^ studied the influence of varying peptide sequence on mechanical and electrical properties and found that varying the length of AA side chains varied the topography, mechanical, and electrical properties of the formed gels. It was demonstrated that the storage modulus could be increased from 3 to 20 kPa, with sheet resistance increasing from 5 to 17 kΩ sq^–1^ and secondary structures varying from α- to β-helix. Varying AA sequences changed the local conformation and stacking of π-conjugates.^370^ Interestingly, the steric effects of the larger aliphatic tails or aromatic groups were found to vary intermolecular electronic coupling within the nanostructures as well as stacking, which was correlated to a reduced sheet resistance.^370^ This demonstrates the tuneability of self-assembled π-conjugated peptides for neural tissue engineering. Thurston et al.^371^ investigated the effect on the electronic properties further through molecular modeling and density functional theory calculations to show that smaller AAs favor linear stacking within the peptide dimer, and this improves the delocalization of electrons. This confirmed that varying the AA sequence changes intermolecular forces and charge transport properties and is therefore an important tool to consider when designing a tissue engineering scaffold for neural regeneration.

**Figure 9:**
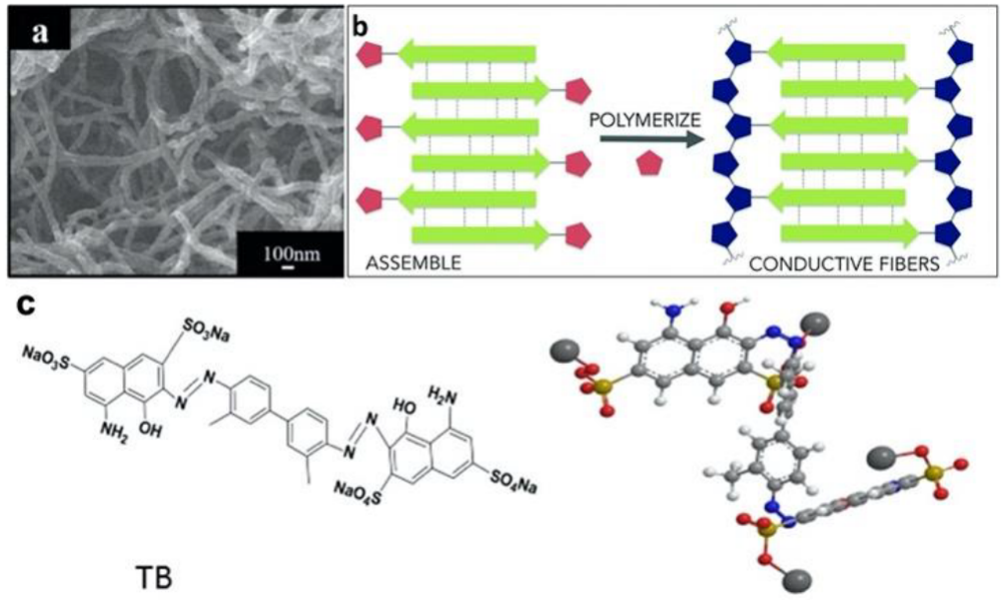
Methods for increasing conductivity of π-conjugated self-assembling systems. (a) SEM of self-doping PPy-TB and (c) Molecular structure of PPy-TB. Reprinted with permission from ref (374). Copyright 2019 American Chemical Society. (b) Schematic of EDOT–OH polymerization along the fiber axis. Reprinted with permission from ref (372). Copyright 2013 American Chemical Society.

Several methods have been explored to enhance the electron transport of self-assembled fibers. Nanofibers have been used as a template to guide the self-assembly of conductive polymers, with additional CP added to the self-assembly dispersion before gelation and oxidation.^362^ The conductive polymer segments can also be covalently cross-linked along the fiber axis to increase conductivity by adding free conductive polymer to the solution. Blatz et al.^372^ explored this idea by functionalizing a peptide with EDOT–OH. The EDOT-modified peptide was polymerized with a 1:3 molar ratio of additional EDOT–OH in organic solvent as shown in Figure 9b.^372^ Conductivity in the order of 10 × 10^–4^ S cm^–1^ were reported, but it was hypothesized that doping the structure could greatly increase its conductivity.^372^ Murphy et al.^364^ synthesized a library of 12 tetrapeptides and functionalized them with EDOT–OH, subsequently polymerizing them with a 1:1 molar ratio and EDOT–OH in aqueous solutions and doped the gel with pTSA.^364^ This yielded gels with conductivities in the order of 1 × 10^–2^ S cm^–1^.^364^ Conductivity of the conjugated systems can also be improved by incorporating dopants within the molecular structure of self-assembling systems.^373^ Yang et al.^374^ created a 3D nanostructured CH for application as a pseudocapacitor. Ionic and electronic conductive properties of PPy and PANI functionalized with different molecules that act as both gelators and dopants were investigated.^374^ These molecules self-assemble into conductive fibers as shown in Figure 9a and can readily form a hydrogel with morphologies dependent on trypan blue (TB) concentrations.^374^ Conductivities as high as 3.3 S/cm were obtained for the PPy-TB molecule shown in Figure 9c.^374^ This hydrogel was designed for energy storage applications, and it is biocompatibility has not been explored but trypan blue is known to be toxic.^375^ However, this is a good demonstration of the use of dopants to increase conductivity in self-assembled π-conjugated systems as other dopants are readily used in the synthesis of CPs for neural tissue engineering.^316^ Another strategy to increase the conductivity is the coassembly of different self-assembling molecules.^376,241^ The coassembly of electron donors and acceptors has been shown to increase conductivity. For example, to create a 1D nanowire that self-assembles in aqueous media, Khalily et al.^377^ coassembled n-type and p-type short peptide-chromophore conjugates. The coassembly showed increased conductivity relative to either the n-type or p-type fibers alone. The n/p-*co*-assembled nanowires are approximately 2400 times more conductive that the n-type wires, and are 10 times more conductive than the p-type alone.^377^

Understanding the relationship between molecular structure and π-stacking is important in the development of conductive self-assembled systems containing π-conjugates. It is therefore interesting to note changes in π-stacking of chromophore self-assemblies. Varying the AA sequence of a PA has also been shown to tune chromophore packing and their resulting photophysics. Tovar et al.^378^ showed that variations of the AA side chain residues at various locations along the AA side chains led to changes in π-stacking. π-stacking has been shown to have profound effects on the conductivity of self-assembled structure metal–organic frameworks,^379,380^ we can therefore infer that conductive peptide packing will be affected by the AA sequence leading to changes in conductivity. This is further reinforced by Lee et al.’s^381^ recent report of very high conductivity in a self-assembly system composed of a π-chromophore core flanked by an alkyl spacer and pentapeptide on both sides. Unexpected mineralization of KCl along the glutamic AA in HCl vapor deposition was obtained following KOH treatment, which is thought to have led to proton doping along with very strong packing and stability.^381^ This method yielded conductivities as high as 1800 S cm^–1^ when incorporating alkyl spacers between the peptides and π-conjugation.^381^ Obtaining such conductivity values demonstrates the potential for the application of molecular self-assembly to form conductive networks.

### 4.2 Switchable SAPs

The uses of electrical stimulation can be targeted not only to neural tissue but also toward specific SAP material features. The possibility of finely tuning the stimulation parameters has promoted the concept of electrically responsive materials.^198−200^ This option confers precise temporal control over material properties and polymerization, fostering the development of cutting-edge applications such as stimuli-responsive drug delivery.^200,206^ This technology provides clear advantages in the field of neural repair, where the sensitive and complex environment requires temporally and spatially precise interventions. Material features such as bioactive cues, wettability, and protein absorption, as well as assembly and disassembly could be controlled by electrical stimulation,^201−204^.^205^ For instance, bioactive biotin molecules can be reversibly exposed depending on the surface potential,^200^ and the presence of an electric voltage can maintain drug-loaded nanoparticles in an assembled configuration to control drug delivery.^206^ It is also known that the natural hydrogel chitosan reversibly self-assembles via electrodeposition of films which are physically cross-linked.^207,208^ Similarly, the redox reaction triggered by electrical stimulation of polydopamine (PDA) coatings have been shown to promote cell spreading, proliferation, and differentiation on a titanium electrode.^209^ This class of materials termed “switchable” can be used to implement highly controllable electrically mediated therapies, allowing for temporal and spatial control of bioactive, topographical, electrochemical, and structural cues to neural cells. Although the properties mentioned are representative of the wide range of possibilities offered by switchable materials, today they only serve as a proof of concept toward an innovative vision for SAP materials.

## 5 Applications

In vivo applications of SAP hydrogels have shown great promise for neural regeneration. Multiple studies have shown that SAPs elicit minimal inflammation and scar formation while promoting vasculature formation, axonal regrowth, and synaptic formation.^19,216^ In the PNS, SAPs have been investigated for treatment of crush and resections of sciatic nerves,^11^ cavernous nerves,^382^^383^ and facial nerves.^219^ Recently, Richter et al.^219^ compared SAP performance to an autograft (using the resected nerve segment) for the regeneration of the facial nerve after a 7.5 mm resection. A hollow collagen tube was filled with an aligned PA and neural regeneration was assessed by electrophysiological stimulation and recording across the nerve resection site.^219^ It was found that the PA showed similar performance to the autograft which is an impressive outcome because autografts are considered the gold standard in treatment.^219^ This PA did not present any bioactive epitopes, but presents evidence that the regenerative potential of SAPs could match autografts and lead to improvements in functional recovery. This is further supported by a 10 mm sciatic nerve resection study led by Yang et al.^384^ Functional recovery similar to that of an autograft was demonstrated by using a SAP functionalized with both IKVAV and RGI, which mimics both laminin and BDNF.^384^

In the CNS treatment of spinal cord injury with SAPs has shown reduced inflammation, cavitation, and scar formation. The promotion of axonal growth and guidance, vascularization, and functional recovery has been observed in animal models.^26,216,224,382,385−388^ In one of the most recent applications to spinal cord regeneration the properties of self-assembling systems were leveraged in order to create a synergistic scaffold. Xiao et al.^389^ have combined topography with bioactivity and drug release. RADA16 SAP containing FGL (neural cell adhesion molecule) was tailored to release Taxol for spinal cord injury in a rat model.^389^ Taxol has been shown useful *in vitro* but has difficulty crossing the BBB and therefore requires localized drug delivery. Following a T9 contusion, the SAPs were injected into the lesion site and rats were evaluated up to 8 weeks after injury. The rats injected with a Taxol-loaded scaffold showed the most functional recovery with a ranking of 15 on the BBB scale, cell infiltration, and neurite extension across the lesion, as well as reduced glial activation and inflammation, and reduced cavity formation.^389^ This is an example of the advantage of tailoring SAPs to control multiple properties critical to regeneration of the nervous system.

SAPs are also an emerging option to accomplish brain regeneration in the context of traumatic brain injury and neurodegenerative diseases.^58,390^ Cell-loaded, drug-loaded, or bioactive injectable SAPs are a strategy for restoring brain tissue and function.^58^ For example, a SAP functionalized with the laminin epitope IKVAV was found to promote the proliferation and differentiation of endogenous NSCs and to improve the learning and memory impairment in an Alzheimer’s disease mice model.^267^

Although there is a plethora of studies on self-assembly systems for neural regeneration both in vivo and in vitro, no complete functional recovery has been observed to date in long peripheral nerve gap injuries or severe central nervous system injuries. Complementary electrical stimulation approaches such as DBS are among the leading treatments for late stage neurodegeneration^14^ and bridging the gap between regeneration therapies and electrical stimulation devices is becoming a necessity.^391^ Smart conductive materials are emerging for bioelectronics applications where the versatility of SAPs could be harnessed for functional electro-neural interface therapies promoting neural regeneration. Chromophore and electroactive peptides have been studied for in vivo drug delivery and tracing and imaging of tumors and have demonstrated good biocompatibility and stability.^178,343,392,393^ However, more work needs to be done on the application of electroactive self-assembling scaffolds for neural regeneration. Material development of biocompatible and electrically tunable SAPs needs to be undertaken with specific attention to biocompatibility of the components and self-assembly mechanism for injection *in vivo*. Additionally, the stability and degradation of electroactive scaffolds in physiological environments need to be understood to characterize its effect on stiffness and electroactivity of the scaffold. The properties of these novel materials can then be tailored to suit the material properties of neural tissue enabling the understanding of the degree of synergistic effects possible through utilizing multiple biomolecules, topographies, and coassemblies. Ultimately the translation of materials developed in the lab not only to *in vivo* models that are known to have regenerative capacity but to the clinic where the systemic patient disease or injury state can impact cell–material interactions needs to be investigated.

## 6 Conclusion

Self-assembling materials provide a significant opportunity for developing next-generation neural regeneration scaffolds. There are challenges in designing injectable biomimetic SAPs, but because of their bottom-up design, these self-assembled structures are highly tailorable. Varying molecular structure and intramolecular interactions, as well as ionic concentrations, can ensure that bioactivity, mechanical, and topographical properties can meet neural tissue engineering regeneration criteria. SAPs offer significant improvement by filling the heterogeneous injury cavities, promoting cell survival, migration, and differentiation as well as axonal growth into through the lesion site. However, in CNS lesions and extensive PNS damage, SAPs do not completely restore function to the injured tissue.

Future developments of SAPs must consider the design of materials that provide a combination of biomimetic cues to promote the regeneration of neural tissue through provision of multiple physical and biochemical cues. Historically, SAPs and indeed other hydrogel materials for neural regeneration have not been tailored through combining all of these biomimetic cues but rather focused on a single or two desired properties. More complex combinatorial systems that better replicate the natural neural milieu may address the current limitations faced by tissue engineering approaches to neural regeneration. Specifically, it is thought that electrical properties will be critical to supporting these electroactive tissues. Various methods exist to incorporate electrical activity into self-assembling systems for the development of nanoelectronics; however, few studies have assessed their application to neural regeneration. Some studies have investigated the application of electroactive or chromophore functionalized self-assembling systems for responsive in vivo drug delivery which suggests the potential biocompatibility of electrically functionalized SAPs. However, key limitations in the current research include minimal characterization of electroactive properties and a lack of understanding of how these components impact degradation and subsequent long-term electroactivity of the scaffold.

## Abbreviations

SAP: self-assembling peptide
ECM: extracellular matrix
CNS: central nervous system
PNS: peripheral nervous system
NSC: neural stem cells
GF: growth factor
NGF: nerve growth factor
BDNF: brain-derived neurotrophic factor
Trk: tyrosine kinase
GAG: glycosamminoglycans
MMP: matrix metalloproteinases
SCI: spinal cord injury
Glc: glycolic acid
NCAM: neural cell adhesion molecule
PA: peptide amphiphile
βFGF: basic-fibroblast cytokine
GDNF: glial cell line derived neurotrophic factor
DBS: deep brain stimulation
FES: functional electrical stimulation
CNT: carbon nanotubes
TB: trypan blue
PDA: polydopamine
